# Supplementary material for: Person-centred care by a combined digital platform and structured telephone support for people with chronic obstructive pulmonary disease and/or chronic heart failure: study protocol for the PROTECT randomised controlled trial
Source: BMJ Open. 2020 Jul 19;10(7):e036356. doi: 10.1136/bmjopen-2019-036356 (PMC7371144; doi:10.1136/bmjopen-2019-036356)
Supplement: Author's manuscript [file bmjopen-2019-036356.draft_revisions.pdf]

BMJ Open is committed to open peer review. As part of this commitment we make the peer review history of every article we publish publicly available.

When an article is published we post the peer reviewers' comments and the authors' responses online. We also post the versions of the paper that were used during peer review. These are the versions that the peer review comments apply to.

The versions of the paper that follow are the versions that were submitted during the peer review process. They are not the versions of record or the final published versions. They should not be cited or distributed as the published version of this manuscript.

BMJ Open is an open access journal and the full, final, typeset and author-corrected version of record of the manuscript is available on our site with no access controls, subscription charges or pay-per-view fees (http://bmjopen.bmj.com).

If you have any questions on BMJ Open's open peer review process please email info.bmjopen@bmj.com

Person-centred care by a combined digital platform and structured telephone support for people with chronic obstructive pulmonary disease and/or chronic heart failure: Study design of the PROTECT randomised controlled trial

Journal:	*BMJ Open*
Manuscript ID	bmjopen-2019-036356
Article Type:	Protocol
Date Submitted by the Author:	12-Dec-2019

Complete List of Authors:	Ali, Lilas; Institute of Health and Care Sciences, Sahlgrenska Academy, University of Gothenburg; University of Gothenburg Centre for Person-Centred Care, Sahlgrenska Academy, University of Gothenburg<br>Wallström, Sara; Institute of Health and Care Sciences, Sahlgrenska Academy, University of Gothenburg; University of Gothenburg Centre for Person-Centred Care, Sahlgrenska Academy, University of Gothenburg<br>Barenfeld, Emmelie; University of Gothenburg Institute of Health and Care Sciences; University of Gothenburg Centre for Person-Centred Care, Sahlgrenska Academy<br>Fors, Andreas; Institute of Health of Care Sciences, Sahlgrenska Academy, University of Gothenburg; Gothenburg Centre for Person-Centred Care (GPCC),<br>Fredholm, Eva; The Swedish Heart & Lung Foundation<br>Gyllensten, Hanna; Health of Care Sciences, Sahlgrenska Academy, University of Gothenburg; Gothenburg Centre for Person-Centred Care (GPCC)<br>Swedberg, Karl; University of Gothenburg, Department of emergency and cardiovascular me; University of Gothenburg Centre for Person-Centred Care, Sahlgrenska Academy<br>Ekman, Inger; Institute of Health and Care Sciences, Sahlgrenska Academy, University of Gothenburg; University of Gothenburg Centre for Person-Centred Care, Sahlgrenska Academy, University of Gothenburg

Keywords:	CARDIOLOGY, Adult cardiology < CARDIOLOGY, Heart failure < CARDIOLOGY, THORACIC MEDICINE

SCHOLARONE™
Manuscripts

I, the Submitting Author has the right to grant and does grant on behalf of all authors of the Work (as defined in the below author licence), an exclusive licence and/or a non-exclusive licence for contributions from authors who are: i) UK Crown employees; ii) where BMJ has agreed a CC-BY licence shall apply, and/or iii) in accordance with the terms applicable for US Federal Government officers or employees acting as part of their official duties; on a worldwide, perpetual, irrevocable, royalty-free basis to BMJ Publishing Group Ltd ("BMJ") its licensees and where the relevant Journal is co-owned by BMJ to the co-owners of the Journal, to publish the Work in this journal and any other BMJ products and to exploit all rights, as set out in our licence.

The Submitting Author accepts and understands that any supply made under these terms is made by BMJ to the Submitting Author unless you are acting as an employee on behalf of your employer or a postgraduate student of an affiliated institution which is paying any applicable article publishing charge ("APC") for Open Access articles. Where the Submitting Author wishes to make the Work available on an Open Access basis (and intends to pay the relevant APC), the terms of reuse of such Open Access shall be governed by a Creative Commons licence – details of these licences and which Creative Commons licence will apply to this Work are set out in our licence referred to above.

Other than as permitted in any relevant BMJ Author's Self Archiving Policies, I confirm this Work has not been accepted for publication elsewhere, is not being considered for publication elsewhere and does not duplicate material already published. I confirm all authors consent to publication of this Work and authorise the granting of this licence.

For peer review only

Person-centred care by a combined digital platform and structured telephone support for people with chronic obstructive pulmonary disease and/or chronic heart failure: Study design of the PROTECT randomised controlled trial.

Lilas Ali[1,2,3], Sara Wallström[1,2], Emmelie Barenfeld[1,2], Andreas Fors[1,2,4],Eva Fredholm [5], Hanna Gyllensten[1,2], Karl Swedberg[1,2,6,7],Inger Ekman[1,2,7]

1 Institute of Health and Care Sciences, Sahlgrenska Academy, University of Gothenburg, Gothenburg, Sweden.

2 Centre for Person-Centred Care (GPCC), University of Gothenburg, Gothenburg, Sweden.

3 Sahlgrenska University Hospital, Psychiatric department, Gothenburg, Sweden.

4 Närhälsan Research and Development Primary Health Care, Region Västra Götaland, Sweden.

5 Patient representative, The Swedish Heart & Lung Foundation.

6 Department of Molecular and Clinical Medicine, Sahlgrenska Academy, University of Gothenburg, Gothenburg, Sweden.

7 Sahlgrenska university hospital/Östra/Department of Internal medicine and geriatrics, Gothenburg, Sweden.

Lilas Ali, Institute of Health Care Sciences, Centre for Person-Centred Care, Sahlgrenska Academy, Box 457, 405 30 Gothenburg, Sweden. lilas.ali@gu.se

For peer review only

Abstract

Introduction: A core feature of chronic obstructive pulmonary disorder (COPD) and chronic heart failure (CHF) is that symptoms may change rapidly because of illness progression. Thus, these chronic conditions are associated with high rehospitalisation rates. Person-centred care (PCC) has been shown to have several benefits for patients with COPD or CHF (or both disorders) but has not yet been investigated through e-health services.

Aim: The project aims to evaluate the effects of PCC by a combined digital platform and structured telephone support for people with chronic obstructive pulmonary disease and/or chronic heart failure.

Methods and analysis: A randomised controlled trial with open, parallel groups that employs a participatory design process will be used. This project will also include process and health economic evaluations of the intervention.

Ethics and dissemination Ethical approval has been secured from the Regional Ethical Review Board in Gothenburg, Sweden (Dnr 063-17 and T063-18). Results will be presented at conferences and to health care professionals, participants and patient organisations. Findings will also be submitted for publication in peer-reviewed journals.

Trial registration: The trial was registered at ClinicalTrials.gov June 7, 2017, identifier: NCT03183817

Keywords: chronic heart failure; chronic obstructive pulmonary disease; e-health; patient-centred care; person-centred care; randomised controlled trial; telehealth.

For peer review only

Article summary

Strengths and limitations of this study

- This project is the first RCT to evaluate the effects of PCC by a combined digital platform and structured telephone support for people with COPD and/ or CHF.
- The protocol details a complex intervention aimed at operationalising person-centred ethics.
- To answer the research questions of the study, collected data will be analysed using quantitative and qualitative methods.
- The evaluation will include patient-reported outcome measures together with health economics and process outcomes.
- Unfortunately, this study did not include individuals without a device for the Internet or those who could not read or write in Swedish.

For peer review only - http://bmjopen.bmj.com/site/about/guidelines.xhtml

Background

Chronic heart failure (CHF) and chronic obstructive pulmonary disease (COPD), two long-term conditions common in older populations, are associated with high rehospitalisation rates and high mortality.[1 2] In addition, both conditions severely affect activities of daily living because of frequent symptoms that limit health-related quality of life (HRQoL), compelling patients to be dependent on others.[3] Patients with CHF and/or COPD frequently require hospital care and the severity of their symptoms often leads to repeated visits to hospital, outpatient or primary care.[1-3] Although pharmacological therapy has improved outcome markedly over the past 10-15 years, management programmes are still needed to optimise care.[4] There are benefits of pulmonary rehabilitation (e.g., reduction in readmission and mortality), including exercise training, education and self-management interventions that specifically target behaviour change for patients with COPD.[5] A consistent recommendation in the treatment of CHF is management programmes designed to improve outcomes through structured follow-ups that increase patients self-care skills and other key issues (e.g., symptoms and disease trajectory).[2] Being affected by a chronic disease with a high symptom burden is a major challenge to living a full and normal life. Health care professionals (HCPs) in conjunction with well-developed technology, can serve to enable successful self-management for individuals with severe long-term illnesses.[6]

Person-centred care

The basis of person-centred care (PCC) is to include the patient's experience, situation, capabilities and resources in the care process. PCC highlights the importance of understanding the person behind the patient as a human being with reason, will, emotions and needs in order for that person to be able to be an active partner in his or her care and

For peer review only - http://bmjopen.bmj.com/site/about/guidelines.xhtml

treatment.[7] Patients (often with the help of relatives) present themselves as persons by expressing their illness narrative. This narrative includes how daily life events are being affected by the condition and treatment and creates a starting point for collaborative care and partnership between HCPs and patients that encourages and empowers patients to actively take part in finding solutions to their problems.[7 8] PCC entails not only identifying health barriers but also to confirm human capabilities and opportunities in the patients' home and local environment. PCC is based on ethical principles, inspired by the capability approach (an alternative approach to welfare economics), which also has been used in research as a theoretical frame of reference in several disciplines (e.g., in economics by the Nobel Laureate Amartya Sen).[9] All people develop their capabilities (such as a sense of responsibility for oneself and others) in relation to other people.

Previous studies evaluating PCC have shown positive effects in patients with several conditions, both acute and long-term, and in different care settings. Positive outcomes include shortened hospital stays,[10] reduced re-admittance,[11] improved self-efficacy,[12] reduced health care costs,[13 14] maintained functional performance,[10] improved HRQoL,[11] improved pain management, greater stamina and enhanced self-reported health,[15] increased physical activity,[16] improved self-efficacy to manage symptoms,[17] reduced fatigue and anxiety[15] and improved experience of the care.[18] Moreover, effects have been especially prominent in patients without post-secondary education,[19] an effect that was sustained for up to 2 years.[20] These studies have applied a PCC approach in practice, wherein the needs of the patient are important but which also incorporates their human ability, which is an often underused resource in health care.[7 21 22]

E-health

For peer review only - http://bmjopen.bmj.com/site/about/guidelines.xhtml

To meet demands on accessible and efficient care one suggested is to facilitate the partnership between HCPs and patients.[23] One such effort can be the care and support provided at a distance through e-health. Today, the Internet is frequently used as a source of information and Sweden belongs to the countries that is high up in a development of maturity of Internet use.[24] Digital web-based care has no geographical constraints, i.e. it is accessible everywhere. It also affords the possibility to reach persons that today cannot be offered traditional treatment or that for other reasons do not dare or want to seek help because they find it stigmatising.[25] The possibility to have contact with HCPs in privacy, when one has the time and when questions arise, is found to be important.[26] Digital health interventions have also been shown to hold promise to improve the quality and experience of cardiovascular care for patients with CHF.[27] Many people seem to determine and manage their illness by themselves, especially when they are concerned about their health. People yearn to be understood and communicate with others with similar experiences.[26] However, previous research has shown that e-health support, in which the users are not directly involved in the process, has low impact. Furthermore, several limitations regarding patients' participation in the design process has been highlighted; it is an anchorage in the home and local environment and opportunities for communication rather than information. Overall, e-health services are considered a promising approach to strengthen self-management among patients with CHF or COPD.[28] E-health interventions that are developed using a participatory design and that uses personal resources are also consistent with the ethics expectations of PCC.

PCC in the form of a structured telephone support for patients with CHF and/ or COPD has been shown to prevent exacerbated self-efficacy without increasing the risk of clinical events.[29] Using a digital symptom tracking tool in combination with PCC has been found to

For peer review only - http://bmjopen.bmj.com/site/about/guidelines.xhtml

improve general self-efficacy compared with standard care.[30] There is, however, a need for studies investigating the effects of PCC via e-health services for people with long-term illness.

Aim

The project aims to evaluate the effects of PCC by a combined digital platform and structured telephone support for people with chronic obstructive pulmonary disease and/or chronic heart failure.

Methods/ design

The design will be a randomised controlled trial (RCT) with open parallel groups. In addition, the project will include process and health economic evaluations of the study. Thus, the project will integrate the collection and analysis of both qualitative and quantitative data to answer the research questions. User experiences and process evaluations are necessary complements to each other to help understand more about enablers and barriers to self-management support using a combined digital platform and telephone support. The protocol complies with the SPIRIT (Standard Protocol Items: Recommendations for Interventional Trials).[31 32]

Patient and public involvement

Public involvement is a cornerstone in PCC. Therefore, this study incorporates a participatory design that assumes that all users (patients, relatives and HCPs) are involved in the study design, which has been reported to facilitate implementation.[33] Extensive consultation with members of the community took place during the development of the digital platform and design of the study. An advisory group consisting of patient

For peer review only

representatives, relatives, HCPs, system-developers and researchers was formed to provide advice and co-design all of the major elements of the study design.

Participants and setting

Study participants will be recruited from nine public primary care centres in Gothenburg that fulfil the inclusion criteria for the study.

Inclusion criteria

- Diagnosed with COPD or CHF
- Listed at one of the nine participating primary care centres
- Must understand written and spoken Swedish

Exclusion criteria

- Not willing to participate
- No device with Internet access, i.e. no computer, smartphone or tablet
- Severe impairment that prevents persons from using e-health support
- No registered address
- Any severe disease with an expected survival of <12 months
- Cognitive impairment
- Ongoing documented diagnosis of alcohol/drug abuse
- Other diseases that can interfere with follow-up (e.g., severe depression, other severe mental illnesses)
- Participating in another conflicting study

Enrolment and randomisation

Designated HCPs will screen medical records of listed patients at the participating primary care centres against study inclusion and exclusion criteria. Eligible patients will be sent a

For peer review only - http://bmjopen.bmj.com/site/about/guidelines.xhtml

letter informing them about the study. The HCPs then contact the eligible patients by phone to give further information about the study and ask whether they willing to participate. If the patient agrees to participate, a consent form together with a return prepaid envelope will be sent to the patient. Once the consent form is returned to the HCPs, patients will be randomised to usual care or PCC plus usual care. The randomisation will be based on a computer-generated list created by a third party. All patients will be informed about group assignments by phone.

Usual care

Patients randomised to usual care will be managed by regular evidence-based treatment and care as outlined in treatment guidelines.[2 5]

Design of intervention

The intervention is based on the theoretical PCC framework in which all people are seen as capable with a unique understanding of themselves and who have unique experiences, expectations, needs, preferences and resources. A central component of PCC is that the professional and patient jointly develop a personal health plan using resources identified in each patient´s illness history as well as defining potential barriers to effective care.[7] Self-efficacy as a concept is closely related to PCC as it also refers to people's belief in their capability to affect daily life events.[7 30 34] In addition, self-efficacy has been shown to have important implications for patient recovery,[35] which is related to the fact that patients confident in their ability to manage everyday life are likely to engage in rehabilitation more actively.[7 34] Steps and components of the intervention are described below.

Description of intervention elements

When the HCPs call the participants in the intervention group to inform them about the outcome of the randomisation procedure, the HCPs will also describe the intervention,

For peer review only - http://bmjopen.bmj.com/site/about/guidelines.xhtml

assist the participants in creating a login to the platform and describe its features, and decide on a date and time for a phone call when the health care plan is created. All the dedicated HCPs participated in the development of the digital platform and received special training in performing PCC communication at a distance. Access to the digital platform will be password protected.

Telephone support and health plan

When the HCPs call the participants, they listen to their narrative about daily life events and how they are affected by the condition, what kind of problems they experience and what resources they have to co-create the health plan. The HCPs try to encourage narration and establish a partnership by using PCC communication skills such as open-ended questions, reflections and summaries. Based on the patient narrative, patient goals, resources and needs are identified and communicated with the patient (participant). The participant (sometimes together with relatives) and the HCPs formulate a person-centred health plan in partnership. The health plan contains five elements:

1. Today we have talked about
2. You would like to be able to do/feel
3. To get there you have to
4. Capabilities and resources that could help you are
5. Support that you might need is

The health plan will be based on mutual agreement between the patient and HCPs and contains the patient's goals, resources and support needs. It also contains follow-up actions and steps needed to achieve progress in the care of the patient. The date of follow-up conversations will be scheduled jointly. This plan will be uploaded to the digital platform

For peer review only - http://bmjopen.bmj.com/site/about/guidelines.xhtml

with help from the HCPs if the participants have chosen to write the plan themselves or it will be written directly by the HCPs and then finalised when accepted by the participants.

The health plan will be the point of departure for the forthcoming conversations and communication via the platform that the participants and the HCPs will have during the study period (of 6 months). The plan will be considered during each patient-HCP contact and revised when necessary. During the intervention, the participants will also be free to contact the HCPs during office hours.

Digital platform

The platform will contain headings such as; messages, daily ratings, health plan, contact, next of kin and links, when logged in that may inspire the patients to make notes on “a good day” respectively “a bad day”. Symptom-ratings and comments will also be made to create a graph over time once the daily ratings have been completed. The HCPs will be able to see the patients´ accounts and make comments. The patients will be able to add or delete any person that they would like to give access to their account. The patients will also be able to customise what content they want visible for invited persons. For instance, they could choose to restrict visibility of the messages between themselves and the HCPs or elect to show all content in their account such as the health plan and daily ratings.

The digital platform will also contain a function to send private messages between the participants and the HCPs. Another function offered in the platform will be the possibility to link relevant information about CHF and COPD that is provided by patient organisations (e.g., the heart and lung association) and the Swedish national help guide (1177.se). There will also be links to a peer-to-peer support group.

Sources and collection of data

For peer review only - http://bmjopen.bmj.com/site/about/guidelines.xhtml

Data will be collected using self-report instruments, interviews, study documentation, medical records and administrative registers. Questionnaires will be mailed (together with a prepaid return envelope) to the participants at study inclusion and 3, 6, 12 and 24 months after randomisation. Baseline data (including demographics, characteristics and medical history) will be collected from the patients' medical records.

Questionnaires

- The General Self-Efficacy Scale (GSE)[36]
- HRQoL (EQ-5D)[37]
- Hospital anxiety and depression scale (HADS)[38]
- Shortness of breath in heart failure (SOB-HF)[39]
- COPD Assessment Test (CAT)[40]
- The Medical Research Council (MRC) breathlessness scale[41]

Primary endpoint

The primary endpoint will be the composite score of change in general self-efficacy and rehospitalisation or death 6 months after randomisation.

A patient is classified as deteriorated if:

- Self-efficacy has decreased by >5 units or
- Patient has been admitted for unscheduled reasons or the patient has died.

A patient is classified as improved if:

- General self-efficacy has increased by >5 units and
- The patient has not been hospitalised or died.

For peer review only - http://bmjopen.bmj.com/site/about/guidelines.xhtml

Those who have neither deteriorated nor improved are considered unchanged.

Secondary endpoints

a. Number of admissions

b. General self-efficacy[36]

c. Health care utilisation measured as the number of admissions and unscheduled outpatient visits due to unplanned visits to hospital or primary care centre due to symptoms of COPD or CHF

d. Hospital Anxiety and Depression Scale(HADS) [38]

e. HRQoL (EQ-5D)[42]

f. Incremental cost-utility ratios

g. Comparing HRQoL and costs for health care

h. CAT[40]

i. The MRC breathlessness scale[41]

Project timeline

Recruitment of participants commenced in August 2017 and was completed in June 2019. During this period, 224 eligible patients had been identified and included in the study. The intervention will continue until early 2020 after which data for the primary outcome will be collected. Data collection for the secondary outcome measures will continue until mid-2021.

Power calculation

To achieve 80% power based on a p-value of 0.05 (two-tailed) for an increase in the proportion of improved patients from 20 to 40% in the primary outcome a sample size of 91

For peer review only

282 patients in each group is required. However, we plan to include 110 patients in each group to have some margin for withdrawals/dropouts. Thus, a minimum of 220 patients will be randomised to the two groups.

285 **Data analysis**

286 Descriptive statistics will be used to characterise the study groups. Between-group differences will be tested using Fisher's exact test for dichotomous variables and parametric and non-parametric tests will be used for continuous variables. Logistic regression will be used to calculate odds ratios with 95% confidence intervals. Statistical significance will be set to $p<0.05$ (two-sided). Both intention-to-treat and per-protocol analyses will be conducted. In addition, subgroup analyses will be performed.

292 **Design of the health economic evaluation**

293 The objective of the health-economic analysis is to estimate differences in total societal costs between the two approaches in relation to the difference in quality-adjusted life-years gained (incremental cost-utility ratios). This analysis will be followed by analysing its dependence on personal characteristics. The estimation includes two steps: a quantify all resource use related to illness and treatment, as well as the HRQoL in physical terms and b) evaluate uses of resources in monetary terms and calculate a utility measure between minus and plus one from the HRQoL instrument [43]. A ratio will also be created that is the incremental cost (the cost difference between intervention and usual care groups) divided by the incremental effect (corresponding difference in utility).

302 Resource use will include data from the regional patient register VEGA (held by Region Västra Götaland), which covers all care reimbursed by the region, including hospital, outpatient and primary care conducted within the region but also care received in other

For peer review only - http://bmjopen.bmj.com/site/about/guidelines.xhtml

regions. Information on prescribed medication and related costs will be obtained from the Swedish Prescribed Drug Register (held by the National Board of Health and Welfare). In addition, data on lost productivity for the patient due to sickness absence (i.e. absence from work that can be attributed to sickness) will be retrieved from the MiDAS database (Social Insurance Agency) and self-report in patient questionnaires (since the first 14 days of each sick-leave are not covered by official statistics). This information will be used to estimate indirect costs (sometimes called productivity costs) using the human capital approach, which entails multiplying the net days on sick leave by the mean wage and associated social security contributions. The difference in costs and utilities will be compared (assuming that there will be no difference in length of life between the two groups) and cost-utility ratios estimated. Several issues are involved in valuing health care in monetary terms related to the basic economic-theoretical concept of "opportunity cost", true societal valuations and marginal costs. We will follow the state of the art practice by making alternative estimates of costs and present the results as sensitivity analyses[44]. Such analyses will be a test of the robustness of the results. HRQoL (measured by EQ-5D) will be collected repeatedly in the patient questionnaires. As a sensitivity analysis, we will use both a general population value set from the UK,[42] as there is no such value set validated for Sweden, and a Swedish experience-based value set[37] for the translation of EQ-5D results into utilities. Missing data from the questionnaires will be considered as missing at random and thus handled using a multiple imputation strategy.

Dependence of personal characteristics (e.g., education and income, collected from the LISA database at Statistics Sweden and through patient questionnaires) will be tested. The distribution of costs will also be analysed by major stakeholders, i.e. county councils/regions, market sectors (productivity loss) and individuals/families/friends to

For peer review only - http://bmjopen.bmj.com/site/about/guidelines.xhtml

facilitate understanding of effects in different parts of society. Both mean effect and variation in the group of participants will be explored.

Design of the process evaluation

A process evaluation will be performed in line with recommendations on how to evaluate complex interventions.[45] The primary objective is to widen and deepen the understanding of intervention mechanisms of impact. Two key questions will guide the evaluation:

- How is the intervention content and design to influence intervention outcomes experienced by people diagnosed with CHF or COPD?
- How are HCPs, other invited partners and people diagnosed with CHF or COPD interacting with a PCC approach that includes an integrated digital platform and structured telephone support system in their everyday life?

A mixed-methods approach will be used to collect data that corresponds with recommendations of how to evaluate complex interventions.[46] Quantitative and qualitative strands will be integrated by adding findings from different studies to understand intervention outcomes and combining methodologies within one study. Patients and next of kin will be interviewed about experiences of the intervention and intervention content and design. HCPs will be interviewed using focus group methodology to obtain a collective understanding[47] followed by individual in-depth interviews. Purposeful sampling will be used and sampling criteria set to reach heterogeneity. The interviews will be recorded and transcribed verbatim. In addition, user data, including which features have been used from the digital platform and ratings of experienced benefits and usability will be collected at the 6- and 12-month follow-ups for all participants randomised to the intervention group and for HCPs. These follow-ups will also include open-ended questions connected to the ratings.

For peer review only - http://bmjopen.bmj.com/site/about/guidelines.xhtml

352 Self-reported use of time will also be collected for participants and health professionals. Qualitative and quantitative strands will be analysed separately using qualitative analysis methods and descriptive statistics. When combining qualitative and quantitative data in the same study, results will be merged into a combined analysis by comparing and matching patterns found in each data source.

357 **Ethical considerations**

358 The study, including the consent forms for participants, was approved by the Regional Ethics Board in Gothenburg (Dnr 063-17 and T6013-18). This study conforms with the declaration of Helsinki.[48] All participants received oral and written informed consent before inclusion in the study.

362 The consent forms used in this study included information to the participants that their data would be treated confidentially and that the results from the study would be published in scientific journals such that individual persons could not be identified.

365 # Discussion

366 This study protocol presents an RCT evaluating the effects of PCC by a combined digital platform and structured telephone support for people with COPD and/or CHF. Previous research has shown that after person-centred telephone support for people with COPD and/or CHF, the level of self-efficacy in the intervention group remained while the control group worsened .[29] However, knowledge about how to promote health in patients with COPD or CHF is limited so research about alternative ways of promoting health is warranted. This study is unique in an e-health context because it is based on PCC, which is an approach that uses patient resources and goals in the planning of care.[7 9] When providing an e-health intervention for older people (≥65 years), one plausible concern is that they might have

For peer review only - http://bmjopen.bmj.com/site/about/guidelines.xhtml

problems in understanding and operating a digital platform. To eliminate this potential issue the HCPs will provide older patients with easy-to-follow instructions on how to create and manage their account. The participants can also contact the HCPs if they need help in managing the account.

In addition, the project intends to evaluate the intervention and identify facilitators and barriers during the process. Complex interventions, such as the one described in this protocol, have multiple interacting components. Consequently, the methods and procedures in such interventions are often less standardised [49] To integrate a process evaluation is therefore highly recommended.[45] A process evaluation provides the opportunity to monitor the intervention and supply information on which parts of the intervention are important and what contextual factors affect the intervention. Evaluation of user experience of the intervention aspires to provide novel knowledge that can be used in clinical practice through an amplified understanding of how the intervention is used and when and for whom it is considered acceptable and beneficial. This novel knowledge can also help to explain programme outcomes.

Interventions must also be cost-effective to be implemented. Accordingly, it is vital to integrate health economic evaluations in the design of a complex intervention. One critique that has been raised against telephone-based interventions is that they may be time-consuming and expensive. However, such interventions have been evaluated and proven cost-effective.[6 50] One example is from our previous study that evaluated PCC using a telephone support programme in which the average time of use was less than 90 minutes per patient during the 6-month study period.[29]

For peer review only

For peer review only - http://bmjopen.bmj.com/site/about/guidelines.xhtml

397 Conducting an intervention with different or more than one diagnosis can cause difficulties in interpreting the findings. However, this is the reality of today's healthcare system. Many older patients have more than one diagnosis and their care situation is often complex.[51] COPD and CHF are both common conditions in older people and they share several symptoms (e.g., fatigue and breathlessness) and often have similar care issues.[52] In addition, comorbidity is common in COPD and CHF.[53] Thus, including patients with both diagnoses is appropriate when reflecting on the challenges and issues facing the healthcare system today.[29]

405 In conclusion, this RCT, which assesses the effects of PCC by an intervention that combines a digital platform with a structured telephone support programme in patients with COPD and/or CHF, will provide clinically relevant information on the effects of PCC on self-reported symptoms, HRQoL and health economics. Our study will also offer information about the barriers and solutions when applying a digital platform together with structured telephone support based on PCC in patients with COPD or CHF.

411 Declarations

412 Authors' affiliations

413 1 Institute of Health and Care Sciences, Sahlgrenska Academy, University of Gothenburg, Gothenburg, Sweden.

415 2 Centre for Person-Centred Care (GPCC), University of Gothenburg, Gothenburg, Sweden.

416 3 Psychiatric Department, Sahlgrenska University Hospital, Gothenburg, Sweden.

417 4 Department of Molecular and Clinical Medicine, Sahlgrenska Academy, University of Gothenburg, Gothenburg, Sweden.

For peer review only - http://bmjopen.bmj.com/site/about/guidelines.xhtml

5 National Heart and Lung Institute, Imperial College, London, United Kingdom.

6 Närhälsan Research and Development Primary Health Care, Region Västra Götaland, Sweden.

Acknowledgements

We want to thank all the participants for sharing their experiences and the participating primary care centres for support and assistance in conducting the study.

Authors' contributions

All authors were involved in the design of the study. LA and SW drafted the manuscript with critical input from EB, AF, HG, EF, KS and IE. IE is the grant holder and project leader. All authors reviewed, edited and approved the final version of the manuscript.

Funding

This work was supported by The Swedish Heart & Lung Foundation (DNr.20180183), the Swedish Research Council (DNr 2017-01230) and the Centre for Person-Centred Care at the University of Gothenburg (GPCC), Sweden.

Competing interests

The authors declare that they have no competing interests.

Patient consent for publication

Because we do not provide personal information in our manuscript, "Consent for publication" is not applicable here.

Data availability statement

The data sets generated and analysed during the current study are not publicly available for ethical reasons but are available from the corresponding author on reasonable request.

For peer review only

For peer review only - http://bmjopen.bmj.com/site/about/guidelines.xhtml

Open access

This is an open access article distributed in accordance with the Creative Commons Attribution Non Commercial (CC BY-NC 4.0) license, which permits others to contribute, remix, adapt, build upon this work non-commercially, and license their derivative works on different terms, provided the original work is properly cited, appropriate credit is given, any changes made indicated and the use is non-commercial (see http://creativecommons.org/licenses/by-nc/4.0/).

Word count

3994

For peer review only

References

1. Rabe KF, Watz H. Chronic obstructive pulmonary disease. *Lancet (London, England)* 2017;389(10082):1931-40. doi: 10.1016/s0140-6736(17)31222-9 [published Online First: 2017/05/18]
2. Ponikowski P, Voors AA, Anker SD, et al. 2016 ESC Guidelines for the Diagnosis and Treatment of Acute and Chronic Heart Failure. *Revista espanola de cardiologia (English ed)* 2016;69(12):1167. doi: 10.1016/j.rec.2016.11.005 [published Online First: 2016/11/30]
3. Theander K, Hasselgren M, Luhr K, et al. Symptoms and impact of symptoms on function and health in patients with chronic obstructive pulmonary disease and chronic heart failure in primary health care. *International journal of chronic obstructive pulmonary disease* 2014;9:785.
4. Holland R, Battersby J, Harvey I, et al. Systematic review of multidisciplinary interventions in heart failure. *Heart* 2005;91(7):899-906.
5. Vogelmeier CF, Criner GJ, Martinez FJ, et al. Global strategy for the diagnosis, management, and prevention of chronic obstructive lung disease 2017 report. GOLD executive summary. *American journal of respiratory and critical care medicine* 2017;195(5):557-82.
6. Wildevuur SE, Simonse LW. Information and communication technology–enabled person-centered care for the "big five" chronic conditions: scoping review. *Journal of medical Internet research* 2015;17(3):e77.
7. Ekman I, Swedberg K, Taft C, et al. Person-centered care—Ready for prime time. *European journal of cardiovascular nursing* 2011;10(4):248-51.
8. Ahmad N, Ellins J, Krelle H, et al. Person-centred care: from ideas to action: Health Foundation 2014.
9. Sen A. Capability and well-being73. *The quality of life* 1993;30
10. Ekman I, Wolf A, Olsson L-E, et al. Effects of person-centred care in patients with chronic heart failure: the PCC-HF study. *European heart journal* 2012;33(9):1112-19.
11. Brännström M, Boman K. Effects of person-centred and integrated chronic heart failure and palliative home care. PREFER: a randomized controlled study. *European journal of heart failure* 2014;16(10):1142-51.
12. Fors A, Ekman I, Taft C, et al. Person-centred care after acute coronary syndrome, from hospital to primary care—a randomised controlled trial. *International journal of cardiology* 2015;187:693-99.
13. Hansson E, Ekman I, Swedberg K, et al. Person-centred care for patients with chronic heart failure - a cost-utility analysis. *European journal of cardiovascular nursing : journal of the Working Group on Cardiovascular Nursing of the European Society of Cardiology* 2016;15(4):276-84. doi: 10.1177/1474515114567035 [published Online First: 2015/01/18]
14. Sahlen KG, Boman K, Brannstrom M. A cost-effectiveness study of person-centered integrated heart failure and palliative home care: Based on a randomized controlled trial. *Palliative medicine* 2016;30(3):296-302. doi: 10.1177/0269216315618544 [published Online First: 2015/11/26]
15. Feldthusen C, Dean E, Forsblad-d'Elia H, et al. Effects of Person-Centered Physical Therapy on Fatigue-Related Variables in Persons With Rheumatoid Arthritis: A Randomized Controlled Trial. *Archives of physical medicine and rehabilitation* 2016;97(1):26-36. doi: 10.1016/j.apmr.2015.09.022 [published Online First: 2015/10/21]
16. Larsson A, Palstam A, Lofgren M, et al. Resistance exercise improves muscle strength, health status and pain intensity in fibromyalgia--a randomized controlled trial. *Arthritis research & therapy* 2015;17:161. doi: 10.1186/s13075-015-0679-1 [published Online First: 2015/06/19]

For peer review only - http://bmjopen.bmj.com/site/about/guidelines.xhtml

17. Fors A, Taft C, Ulin K, et al. Person-centred care improves self-efficacy to control symptoms after acute coronary syndrome: a randomized controlled trial. *European Journal of Cardiovascular Nursing* 2016;15(2):186-94.
18. Wolf A, Vella R, Fors A. The impact of person-centred care on patients' care experiences in relation to educational level after acute coronary syndrome: secondary outcome analysis of a randomised controlled trial. *European journal of cardiovascular nursing : journal of the Working Group on Cardiovascular Nursing of the European Society of Cardiology* 2019;18(4):299-308. doi: 10.1177/1474515118821242 [published Online First: 2019/01/18]
19. Fors A, Gyllensten H, Swedberg K, et al. Effectiveness of person-centred care after acute coronary syndrome in relation to educational level: Subgroup analysis of a two-armed randomised controlled trial. *International journal of cardiology* 2016;221:957-62.
20. Fors A, Swedberg K, Ulin K, et al. Effects of person-centred care after an event of acute coronary syndrome: Two-year follow-up of a randomised controlled trial. *Int J Cardiol* 2017;249:42-47. doi: 10.1016/j.ijcard.2017.08.069 [published Online First: 2017/09/13]
21. Ekman I, Hedman H, Swedberg K, et al. Commentary: Swedish initiative on person centred care. *BMJ (Clinical research ed)* 2015;350:h160. doi: 10.1136/bmj.h160 [published Online First: 2015/02/12]
22. Jansson I, Fors A, Ekman I, et al. Documentation of person-centred health plans for patients with acute coronary syndrome. *European journal of cardiovascular nursing : journal of the Working Group on Cardiovascular Nursing of the European Society of Cardiology* 2018;17(2):114-22. doi: 10.1177/1474515117718415 [published Online First: 2017/06/29]
23. Sanders EB-N, Stappers PJ. Co-creation and the new landscapes of design. *Co-design* 2008;4(1):5-18.
24. Foundation TSI. The swedes and the internet 2019.: Internet Stiftelsen, 2019.
25. Erdelez S, Tanacković SF, Balog KP. Online behavior of the alzheimer's disease patient caregivers on croatian online discussion forum. *Proceedings of the Association for Information Science and Technology* 2019;56(1):78-88.
26. Ali L, Fors A, Ekman I. Need of support in people with chronic obstructive pulmonary disease. *Journal of clinical nursing* 2017
27. Frederix I, Caiani EG, Dendale P, et al. ESC e-Cardiology Working Group Position Paper: Overcoming challenges in digital health implementation in cardiovascular medicine. *European journal of preventive cardiology* 2019;26(11):1166-77.
28. Hanlon P, Daines L, Campbell C, et al. Telehealth Interventions to Support Self-Management of Long-Term Conditions: A Systematic Metareview of Diabetes, Heart Failure, Asthma, Chronic Obstructive Pulmonary Disease, and Cancer. *J Med Internet Res* 2017;19(5):e172. doi: 10.2196/jmir.6688 [published Online First: 2017/05/21]
29. Fors A, Blanck E, Ali L, et al. Effects of a person-centred telephone-support in patients with chronic obstructive pulmonary disease and/or chronic heart failure–A randomized controlled trial. *PloS one* 2018;13(8):e0203031.
30. Wolf A, Fors A, Ulin K, et al. An eHealth Diary and Symptom-Tracking Tool Combined With Person-Centered Care for Improving Self-Efficacy After a Diagnosis of Acute Coronary Syndrome: A Substudy of a Randomized Controlled Trial. *J Med Internet Res* 2016;18(2):e40. doi: 10.2196/jmir.4890 [published Online First: 2016/02/26]
31. Chan AW, Tetzlaff JM, Altman DG, et al. SPIRIT 2013 Statement: defining standard protocol items for clinical trials. *Revista panamericana de salud publica = Pan American journal of public health* 2015;38(6):506-14. [published Online First: 2016/07/22]
32. Chan AW, Tetzlaff JM, Gotzsche PC, et al. SPIRIT 2013 explanation and elaboration: guidance for protocols of clinical trials. *BMJ (Clinical research ed)* 2013;346:e7586. doi: 10.1136/bmj.e7586 [published Online First: 2013/01/11]
33. Spinuzzi C. The methodology of participatory design. *Technical Communication* 2005;52(2):163-74.

For peer review only

For peer review only - http://bmjopen.bmj.com/site/about/guidelines.xhtml

34. Bandura A. Self-efficacy: toward a unifying theory of behavioral change. *Psychological review* 1977;84(2):191.
35. Lorig KR, Ritter P, Stewart AL, et al. Chronic disease self-management program: 2-year health status and health care utilization outcomes. *Medical care* 2001;39(11):1217-23. doi: 10.1097/00005650-200111000-00008 [published Online First: 2001/10/19]
36. Schwarzer R, Jerusalem M. Generalized self-efficacy scale. *Measures in health psychology: A user's portfolio Causal and control beliefs* 1995;1(1):35-37.
37. Burstrom K, Sun S, Gerdtham UG, et al. Swedish experience-based value sets for EQ-5D health states. *Quality of life research : an international journal of quality of life aspects of treatment, care and rehabilitation* 2014;23(2):431-42. doi: 10.1007/s11136-013-0496-4 [published Online First: 2013/08/27]
38. Zigmond AS, Snaith RP. The hospital anxiety and depression scale. *Acta psychiatrica scandinavica* 1983;67(6):361-70.
39. Ekman I, Granger B, Swedberg K, et al. Measuring shortness of breath in heart failure (SOB-HF): development and validation of a new dyspnoea assessment tool. *European journal of heart failure* 2011;13(8):838-45.
40. Jones P, Brusselle G, Dal Negro R, et al. Properties of the COPD assessment test in a cross-sectional European study. *European Respiratory Journal* 2011;38(1):29-35.
41. Stenton C. The MRC breathlessness scale. *Occupational Medicine* 2008;58(3):226-27.
42. Dolan P, Gudex C, Kind P, et al. A social tariff for EuroQol: results from a UK general population survey, 1995.
43. Segel JE. Cost-of-illness studies—a primer. *RTI-UNC Center of Excellence in Health Promotion Economics* 2006:1-39.
44. Drummond MF, Drummond MF, McGuire A. Economic evaluation in health care: merging theory with practice: OUP Oxford 2001.
45. Moore GF, Audrey S, Barker M, et al. Process evaluation of complex interventions: Medical Research Council guidance. *BMJ (Clinical research ed)* 2015;350:h1258.
46. Craig P, Dieppe P, Macintyre S, et al. Developing and evaluating complex interventions: the new Medical Research Council guidance. *BMJ (Clinical research ed)* 2008;337:a1655. doi: 10.1136/bmj.a1655 [published Online First: 2008/10/01]
47. Krueger RA. Focus groups: A practical guide for applied research: Sage publications 2014.
48. World Medical Association Declaration of Helsinki: ethical principles for medical research involving human subjects. *Jama* 2013;310(20):2191-4. doi: 10.1001/jama.2013.281053 [published Online First: 2013/10/22]
49. Campbell NC, Murray E, Darbyshire J, et al. Designing and evaluating complex interventions to improve health care. *BMJ (Clinical research ed)* 2007;334(7591):455-59.
50. Inglis SC, Clark RA, Dierckx R, et al. Structured telephone support or non-invasive telemonitoring for patients with heart failure. *Cochrane Database of Systematic Reviews* 2015(10)
51. Alfredsson J, Alexander KP. Multiple chronic conditions in older adults with acute coronary syndromes. *Clinics in geriatric medicine* 2016;32(2):291-303.
52. Dharmarajan K, Strait KM, Tinetti ME, et al. Treatment for multiple acute cardiopulmonary conditions in older adults hospitalized with pneumonia, chronic obstructive pulmonary disease, or heart failure. *Journal of the American Geriatrics Society* 2016;64(8):1574-82.
53. Hawkins NM, Petrie MC, Jhund PS, et al. Heart failure and chronic obstructive pulmonary disease: diagnostic pitfalls and epidemiology. *Eur J Heart Fail* 2009;11(2):130-9. doi: 10.1093/eurjhf/hfn013 [published Online First: 2009/01/27]

For peer review only

For peer review only - http://bmjopen.bmj.com/site/about/guidelines.xhtml

SPIRIT Checklist

Item	Page
1	1
2a	2
2b	N/A
3	N/A
4	20
5a	19,20
5b	N/A
5c	20
5d	N/A
6a	4-7
6b	N/A
7	7
8	7-17
9	8
10	8
11a	9-12
11b	N/A
11c	N/A
11d	9
12	12-13
13	13
14	13-14
15	13-14
16a	8-9
16b	8-9
16c	8-9
17a	7
17b	N/A
18a	7-17
18b	11-13
19	N/A
20a	14
20b	14
20c	14
21a	N/A
21b	N/A
22	N/A
23	N/A
24	17
25	N/A
26a	8-9

For peer review only

26b	N/A
27	17
28	20
29	20
30	N/A
31a	2
31b	N/A
31c	20

For peer review only

BMJ Open

Person-centred care by a combined digital platform and structured telephone support for people with chronic obstructive pulmonary disease and/or chronic heart failure: Study design of the PROTECT randomised controlled trial

Journal:	*BMJ Open*
Manuscript ID	bmjopen-2019-036356.R1
Article Type:	Protocol
Date Submitted by the Author:	27-Feb-2020
Complete List of Authors:	Ali, Lilas; Institute of Health and Care Sciences, Sahlgrenska Academy, University of Gothenburg; University of Gothenburg Centre for Person-Centred Care, Sahlgrenska Academy, University of Gothenburg<br>Wallström, Sara; Institute of Health and Care Sciences, Sahlgrenska Academy, University of Gothenburg; University of Gothenburg Centre for Person-Centred Care, Sahlgrenska Academy, University of Gothenburg<br>Barenfeld, Emmelie; University of Gothenburg Institute of Health and Care Sciences; University of Gothenburg Centre for Person-Centred Care, Sahlgrenska Academy<br>Fors, Andreas; Institute of Health of Care Sciences, Sahlgrenska Academy, University of Gothenburg; Gothenburg Centre for Person-Centred Care (GPCC),<br>Fredholm, Eva; The Swedish Heart & Lung Foundation<br>Gyllensten, Hanna; Health of Care Sciences, Sahlgrenska Academy, University of Gothenburg; Gothenburg Centre for Person-Centred Care (GPCC)<br>Swedberg, Karl; University of Gothenburg, Department of emergency and cardiovascular me; University of Gothenburg Centre for Person-Centred Care, Sahlgrenska Academy<br>Ekman, Inger; Institute of Health and Care Sciences, Sahlgrenska Academy, University of Gothenburg; University of Gothenburg Centre for Person-Centred Care, Sahlgrenska Academy, University of Gothenburg
**Primary Subject Heading**:	Nursing
Secondary Subject Heading:	Cardiovascular medicine, General practice / Family practice, Health economics, Nursing, Respiratory medicine
Keywords:	CARDIOLOGY, Adult cardiology < CARDIOLOGY, Heart failure < CARDIOLOGY, THORACIC MEDICINE

SCHOLARONE™
Manuscripts

I, the Submitting Author has the right to grant and does grant on behalf of all authors of the Work (as defined in the below author licence), an exclusive licence and/or a non-exclusive licence for contributions from authors who are: i) UK Crown employees; ii) where BMJ has agreed a CC-BY licence shall apply, and/or iii) in accordance with the terms applicable for US Federal Government officers or employees acting as part of their official duties; on a worldwide, perpetual, irrevocable, royalty-free basis to BMJ Publishing Group Ltd ("BMJ") its licensees and where the relevant Journal is co-owned by BMJ to the co-owners of the Journal, to publish the Work in this journal and any other BMJ products and to exploit all rights, as set out in our licence.

The Submitting Author accepts and understands that any supply made under these terms is made by BMJ to the Submitting Author unless you are acting as an employee on behalf of your employer or a postgraduate student of an affiliated institution which is paying any applicable article publishing charge ("APC") for Open Access articles. Where the Submitting Author wishes to make the Work available on an Open Access basis (and intends to pay the relevant APC), the terms of reuse of such Open Access shall be governed by a Creative Commons licence – details of these licences and which Creative Commons licence will apply to this Work are set out in our licence referred to above.

Other than as permitted in any relevant BMJ Author's Self Archiving Policies, I confirm this Work has not been accepted for publication elsewhere, is not being considered for publication elsewhere and does not duplicate material already published. I confirm all authors consent to publication of this Work and authorise the granting of this licence.

For peer review only

200226

Person-centred care by a combined digital platform and structured telephone support for people with chronic obstructive pulmonary disease and/or chronic heart failure: Study design of the PROTECT randomised controlled trial.

Lilas Ali[1,2,3], Sara Wallström[1,2], Emmelie Barenfeld[1,2], Andreas Fors[1,2,4],Eva Fredholm [5], Hanna Gyllensten[1,2], Karl Swedberg[1,2,6,7],Inger Ekman[1,2,7]

1 Institute of Health and Care Sciences, Sahlgrenska Academy, University of Gothenburg, Gothenburg, Sweden.

2 Centre for Person-Centred Care (GPCC), University of Gothenburg, Gothenburg, Sweden.

3 Sahlgrenska University Hospital, Psychiatric department, Gothenburg, Sweden.

4 Närhälsan Research and Development Primary Health Care, Region Västra Götaland, Sweden.

5 Patient representative, The Swedish Heart & Lung Foundation.

6 Department of Molecular and Clinical Medicine, Sahlgrenska Academy, University of Gothenburg, Gothenburg, Sweden.

7 Sahlgrenska university hospital/Östra/Department of Internal medicine and geriatrics, Gothenburg, Sweden.

Lilas Ali, Institute of Health Care Sciences, Centre for Person-Centred Care, Sahlgrenska Academy, Box 457, 405 30 Gothenburg, Sweden. lilas.ali@gu.se

For peer review only

For peer review only - http://bmjopen.bmj.com/site/about/guidelines.xhtml

200226

Abstract

Background: A core feature of chronic obstructive pulmonary disorder (COPD) and chronic heart failure (CHF) is that symptoms may change rapidly because of illness progression. Thus, these chronic conditions are associated with high rehospitalisation rates. Person-centred care (PCC) has been shown to have several benefits for patients with COPD or CHF (or both disorders) but has not yet been investigated through e-health services.

Aim: The project aims to evaluate the effects of PCC by a combined digital platform and structured telephone support for people with chronic obstructive pulmonary disease and/or chronic heart failure.

Methods and analysis: A randomised controlled trial with open, parallel groups that employs a participatory design process will be used. This project will also include process and health economic evaluations of the intervention.

Ethics and dissemination Ethical approval has been secured from the Regional Ethical Review Board in Gothenburg, Sweden (Dnr 063-17 and T063-18). Results will be presented at conferences and to health care professionals, participants and patient organisations. Findings will also be submitted for publication in peer-reviewed journals.

Trial registration: The trial was registered at ClinicalTrials.gov June 7, 2017, identifier: NCT03183817

Keywords: chronic heart failure; chronic obstructive pulmonary disease; e-health; patient-centred care; person-centred care; randomised controlled trial; telehealth.

For peer review only - http://bmjopen.bmj.com/site/about/guidelines.xhtml

200226

Article summary

Strengths and limitations of this study

- This project is the first RCT to evaluate the effects of PCC by a combined digital platform and structured telephone support for people with COPD and/ or CHF.
- The protocol details a complex intervention aimed at operationalising person-centred ethics.
- To answer the research questions of the study, collected data will be analysed using quantitative and qualitative methods.
- The evaluation will include patient-reported outcome measures together with health economics and process outcomes.
- Unfortunately, this study did not include individuals without a device for the Internet or those who could not read or write in Swedish.

For peer review only

200226

Introduction

Chronic heart failure (CHF) and chronic obstructive pulmonary disease (COPD), two long-term conditions common in older populations, are associated with high rehospitalisation rates and high mortality.[1 2] In addition, both conditions severely affect activities of daily living because of frequent symptoms that limit health-related quality of life (HRQoL), compelling patients to be dependent on others.[3] Patients with CHF and/or COPD frequently require hospital care and the severity of their symptoms often leads to repeated visits to hospital, outpatient or primary care.[1-3] Although pharmacological therapy has improved outcomes markedly over the past 10-15 years, management programmes are still needed to optimise care.[4] There are benefits of pulmonary rehabilitation (e.g., reduction in readmission and mortality), including exercise training, education and self-management interventions that specifically target behaviour change for patients with COPD.[5] A consistent recommendation in the treatment of CHF is management programmes designed to improve outcomes through structured follow-ups that increase patients´ self-care skills and other key issues (e.g., symptoms and disease trajectory).[2] Being affected by a chronic disease with a high symptom burden is a major challenge to living a full life. Health care professionals (HCPs), such as registered nurses, occupational therapist and physiotherapist, in conjunction with well-developed technology, can serve to enable successful self-management for individuals with severe long-term illnesses.[6]

Person-centred care

The basis of person-centred care (PCC) is to include the patient's experience, situation, capabilities and resources in the care process. PCC highlights the importance of understanding the person behind the patient as a human being with reason, will, emotions

For peer review only

200226

and needs in order for that person to be able to be an active partner in his or her care and treatment.[7] Patients (often with the help of relatives) present themselves as persons by expressing their illness narrative. This narrative includes how daily life events are being affected by the condition and treatment and creates a starting point for collaborative care and partnership between HCPs and patients that encourages and empowers patients to actively take part in finding solutions to their problems.[7 8] PCC entails not only identifying health barriers but also to confirm human capabilities and opportunities in the patients' home and local environment. PCC is based on ethical principles, inspired by the capability approach (an alternative approach to welfare economics), which also has been used in research as a theoretical frame of reference in several disciplines (e.g., in economics by the Nobel Laureate Amartya Sen).[9] All people develop their capabilities (such as a sense of responsibility for oneself and others) in relation to other people.

Previous studies evaluating PCC that for example utilizes personal health plans and symptom tracking have shown positive effects in patients with several conditions, both acute and long-term, and in different care settings. Positive outcomes include shortened hospital stays,[10] reduced re-admittance,[11] improved self-efficacy,[12] reduced health care costs,[13 14] maintained functional performance,[10] improved HRQoL,[11] improved pain management, greater stamina and enhanced self-reported health,[15] increased physical activity,[16] improved self-efficacy to manage symptoms,[17] reduced fatigue and anxiety[15] and improved experience of the care.[18] Moreover, effects have been especially prominent in patients without post-secondary education,[19] an effect that was sustained for up to 2 years.[20] These studies have applied a PCC approach in practice, wherein the needs of the patient are important but which also incorporates their human ability, which is an often underused resource in health care.[7 21 22]

For peer review only - http://bmjopen.bmj.com/site/about/guidelines.xhtml

200226

E-health

To meet demands on accessible and efficient care one suggestion is to facilitate the partnership between HCPs and patients.[23] One such effort can be the care and support provided through e-health. Today, the Internet is frequently used as a source of information and Sweden belongs to the countries that is high up in a development of maturity of Internet use concerning the number of computers and the possibility of accessing the Internet.[24] Digital web-based care has no geographical constraints, i.e. it is potentially accessible everywhere. It also affords the possibility to reach persons that today avoids to seek help because they find it stigmatising.[25] Moreover, the possibility of having contact with HCPs in privacy, when one has the time and when questions arise, is found to be important.[26] Many people want to manage their health by themselves, especially when they are concerned about their illness. Yet people yearn to be understood and communicate with others with similar experiences.[26] E-health solutions offer these possibilities. Digital health interventions have also been shown to hold promise to improve the quality and experience of care for patients with CHF.[27]

There are, however, some concerns that need to be taken into account when designing e-health solutions. There are some indications that older people can find e-health solutions difficult to handle.[28]Previous research have also shown that e-health support, in which the users are not directly involved in the process, has low impact.[29]

There is some research combining e-health with PCC. PCC in the form of a structured telephone support for patients with CHF and/ or COPD has been shown to mitigate worsening self-efficacy without increasing the risk of clinical events.[30] Using a digital symptom tracking tool in combination with PCC has been found to improve general self-

For peer review only - http://bmjopen.bmj.com/site/about/guidelines.xhtml

200226

efficacy compared with standard care.[31] E-health interventions that are developed using a participatory design and that uses personal resources are also consistent with the ethics expectations of PCC. Overall, e-health solutions are considered a promising approach to strengthen self-management among patients with CHF or COPD.[29] There is, however, a need for studies investigating the effects of PCC via e-health solutions for people with long-term illness.

Aim

The project aims to evaluate the effects of PCC by a combined digital platform and structured telephone support for people with chronic obstructive pulmonary disease and/or chronic heart failure.

Methods/ design

The design will be a randomised controlled trial (RCT) with open parallel groups. In addition, the project will include process and health economic evaluations of the study. Thus, the project will integrate the collection and analysis of both qualitative and quantitative data to answer the research questions. User experiences and process evaluations are necessary complements to each other to help understand more about enablers and barriers to self-management support using a combined digital platform and telephone support. The protocol complies with the SPIRIT (Standard Protocol Items: Recommendations for Interventional Trials).[32 33]

Patient and public involvement

Public involvement is a cornerstone in PCC. Therefore, this study incorporates a participatory design that assumes that all users (patients, relatives and HCPs) are involved in the study design, which has been reported to facilitate implementation.[34] Extensive

For peer review only - http://bmjopen.bmj.com/site/about/guidelines.xhtml
For peer review only

consultation with members of the community took place during the development of the digital platform and design of the study. During these consultations the patient representatives requested that the platform should include functionalities such as ability for two-way communication, reliable information and advice about their condition and that it should be user friendly. The HCPs contributed with input concerning layout and technical functionalities of the platform. An advisory group consisting of patient representatives, relatives, HCPs, system-developers and researchers was formed to provide advice and co-design all of the major elements of the study design

Participants and setting

Study participants that fulfil the inclusion criteria of the study will be recruited from nine public primary care centres in Gothenburg.

Inclusion criteria

- Diagnosed with COPD or CHF
- Listed at one of the nine participating primary care centres
- Must understand written and spoken Swedish

Exclusion criteria

- No device with Internet access, i.e. no computer, smartphone or tablet
- Severe impairment that prevents persons from using e-health support
- No registered address
- Any severe disease with an expected survival of <12 months
- Cognitive impairment
- Ongoing documented diagnosis of alcohol/drug abuse

For peer review only

200226

- Other diseases that can interfere with follow-up (e.g., severe depression, other severe mental illnesses)
- Participating in another conflicting study

Enrolment and randomisation

Designated HCPs will screen medical records of listed patients at the participating primary care centres against study inclusion and exclusion criteria. Eligible patients will be sent a letter informing them about the study. The HCPs then contact the eligible patients by phone to give further information about the study and ask whether they willing to participate. If the patient agrees to participate, a consent form together with a return prepaid envelope will be sent to the patient. Once the consent form is returned to the HCPs, patients will be randomised to usual care or PCC plus usual care. The randomisation will be based on a computer-generated list created by a third party. All patients will be informed about group assignments by phone.

Usual care

Patients randomised to usual care will be managed by regular evidence-based treatment and care as outlined in treatment guidelines.[2 5]

Design of intervention

The intervention is based on the theoretical PCC framework in which all people are seen as capable with a unique understanding of themselves and who have unique experiences, expectations, needs, preferences and resources. A central component of PCC is that the professional and patient jointly develop a personal health plan using resources identified in each patient´s illness history as well as defining potential barriers to effective care.[7] Self-efficacy as a concept is closely related to PCC as it also refers to people's belief in their capability to affect daily life events.[7 31 35] In addition, self-efficacy has been shown to have

For peer review only - http://bmjopen.bmj.com/site/about/guidelines.xhtml

200226

important implications for patient recovery,[36] which is related to the fact that patients confident in their ability to manage everyday life are likely to engage in rehabilitation more actively.[7 35] Steps and components of the intervention are described below.

Description of intervention elements

When the HCPs call the participants in the intervention group to inform them about the outcome of the randomisation procedure, the HCPs will also describe the intervention, assist the participants in creating a login to the platform and describe its features, and decide on a date and time for a phone call when the health care plan is created. All the dedicated HCPs participated in the development of the digital platform and received special training in performing PCC communication at a distance. Access to the digital platform will be password protected.

Telephone support and health plan

When the HCPs call the participants, they listen to their narrative about daily life events and how they are affected by the condition, what kind of problems they experience and what resources they have to co-create the health plan. The HCPs try to encourage narration and establish a partnership by using PCC communication skills such as open-ended questions, reflections and summaries. Based on the patient narrative, patient goals, resources and needs are identified and communicated with the patient (participant). The participant (sometimes together with relatives) and the HCPs formulate a person-centred health plan in partnership. The health plan contains five elements:

1. Today we have talked about
2. You would like to be able to do/feel
3. To get there you have to

For peer review only

200226

4. Capabilities and resources that could help you are
5. Support that you might need is

The health plan will be based on mutual agreement between the patient and HCPs and contains the patient's goals, resources and support needs. It also contains follow-up actions and steps needed to achieve progress in the care of the patient. The date of follow-up conversations will be scheduled jointly. This plan will be uploaded to the digital platform with help from the HCPs if the participants have chosen to write the plan themselves or it will be written directly by the HCPs and then finalised when accepted by the participants.

The health plan will be the point of departure for the forthcoming conversations and communication via the platform that the participants and the HCPs will have during the study period (of 6 months). The plan will be considered during each follow-up when the HCPs contact the participants and revised when necessary. During the intervention, the participants will also be free to contact the HCPs during office hours.

Digital platform

The platform will contain headings such as; messages, daily ratings, health plan, contact, next of kin and links, when logged in that may inspire the patients to make notes on "a good day" respectively "a bad day". Functionalities of the platform include ability for two-way communication, possibility to rate symptoms and a collection of links to information concerning COPD and CHF. Symptom-ratings and comments will also be made to create a graph over time once the daily ratings have been completed. The HCPs will be able to see the patients´ accounts and make comments. The patients will be able to add or delete any person (for example informal carers, family or friends) that they would like to give access to their account. The patients will also be able to customise what content they want visible for

For peer review only - http://bmjopen.bmj.com/site/about/guidelines.xhtml

invited persons. For instance, they could choose to restrict visibility of the messages between themselves and the HCPs or elect to show all content in their account such as the health plan and daily ratings.

The digital platform will also contain a function to send private messages between the participants and the HCPs. The HCPs check for new messages daily. Another function offered in the platform will be the possibility to link relevant information about CHF and COPD that is provided by patient organisations (e.g., the heart and lung association) and the Swedish national help guide (1177.se). There will also be links to a peer-to-peer support group.

Sources and collection of data

Data will be collected using self-report instruments, interviews, study documentation, medical records and administrative registers. Questionnaires will be mailed (together with a prepaid return envelope) to the participants at study inclusion and 3, 6, 12 and 24 months after randomisation. Paper questionnaires were chosen since not all of the instruments are validated for online use. In connection with the follow-ups and adverse events (i.e. admission or death) will be controlled for all participants. Baseline data (including demographics, characteristics and medical history) will be collected from the patients' medical records. For details, see Figure 1.

Questionnaires

- The General Self-Efficacy Scale (GSE)[37]
- EuroQol-5 Dimensions health state questionnaire (EQ-5D)[38]
- Hospital Anxiety and Depression Scale (HADS)[39]
- Shortness of Breath in Heart Failure (SOB-HF)[40]

For peer review only - http://bmjopen.bmj.com/site/about/guidelines.xhtml

- COPD Assessment Test (CAT)[41]
- The Medical Research Council (MRC) breathlessness scale[42]

Primary endpoint

The primary endpoint will be the composite score of change in general self-efficacy and rehospitalisation or death 6 months after randomisation.

A patient will be classified as deteriorated if:

- general self-efficacy has decreased by >5 units or
- patient has been admitted for unscheduled reasons due to COPD/ CHF or
- patient has died due to all causes.

A patient is classified as improved if:

- general self-efficacy has increased by >5 units and
- the patient has not been hospitalised due to COPD/CHF and
- the patient has not died.

Those who have neither deteriorated nor improved are considered unchanged.

Secondary endpoints

a. Number of admissions

b. General self-efficacy[37]

c. Health care utilisation measured as the number of admissions and unscheduled outpatient visits due to unplanned visits to hospital or primary care centre due to symptoms of COPD or CHF

For peer review only

For peer review only - http://bmjopen.bmj.com/site/about/guidelines.xhtml

200226

d. HADS [39]

e. HRQoL (EQ-5D)[43]

f. Incremental cost-utility ratios

g. Comparing HRQoL and costs for health care

h. CAT[41]

i. The MRC breathlessness scale[42]

j. SOB-HF[40]

Project timeline

Recruitment of participants commenced in August 2017 and was completed in June 2019. During this period, 224 eligible patients had been identified and included in the study. The intervention will continue until early 2020 after which data for the primary outcome will be collected. Data collection for the secondary outcome measures will continue until mid-2021.

Power calculation

To achieve 80% power based on a p-value of 0.05 (two-tailed) for an increase in the proportion of improved patients from 20 to 40% in the primary outcome a sample size of 91 patients in each group is required. However, we plan to include 110 patients in each group to have some margin for withdrawals/dropouts. Thus, a minimum of 220 patients will be randomised across the two groups.

Quantitative data analysis

Descriptive statistics will be used to characterise the study groups. Between-group differences will be tested using Fisher's exact test for dichotomous variables and parametric and non-parametric tests will be used for continuous variables. Logistic regression will be

For peer review only

For peer review only - http://bmjopen.bmj.com/site/about/guidelines.xhtml

200226

used to calculate odds ratios with 95% confidence intervals. Statistical significance will be set to $p<0.05$ (two-sided). Both intention-to-treat and per-protocol analyses will be conducted. In addition, subgroup analyses will be performed.

Design of the health economic evaluation

The objective of the health-economic analysis is to estimate differences in total societal costs between the two approaches in relation to the difference in quality-adjusted life-years gained (incremental cost-utility ratios) over the two-year follow-up. This analysis will be followed by analysing its dependence on personal characteristics. The estimation includes two steps: a quantify all resource use related to illness and treatment, as well as the HRQoL in physical terms and b) evaluate uses of resources in monetary terms and calculate a utility measure between minus and plus one from the HRQoL instrument [44]. A ratio will also be created that is the incremental cost (the cost difference between intervention and usual care groups) divided by the incremental effect (corresponding difference in utility).

Resource use will include data from the regional patient register VEGA (held by Region Västra Götaland), which covers all care reimbursed by the region, including hospital, outpatient and primary care conducted within the region but also care received in other regions. Information on prescribed medication and related costs will be obtained from the National Board of Health and Welfare. In addition, data on lost productivity for the patient due to sickness absence (i.e. absence from work that can be attributed to sickness) will be retrieved from the MiDAS database (Social Insurance Agency) and self-report in patient questionnaires (since the first 14 days of each sick-leave are not covered by official statistics). This information will be used to estimate indirect costs (sometimes called productivity costs) using the human capital approach, which entails multiplying the net days

For peer review only - http://bmjopen.bmj.com/site/about/guidelines.xhtml

200226

on sick leave by the mean wage and associated social security contributions. The difference in costs and utilities will be compared (assuming that there will be no difference in length of life between the two groups) and cost-utility ratios estimated. Several issues are involved in valuing health care in monetary terms related to the basic economic-theoretical concept of "opportunity cost", true societal valuations and marginal costs. We will follow the state of the art practice by making alternative estimates of costs and present the results as sensitivity analyses[45]. Such analyses will be a test of the robustness of the results. HRQoL (measured by EQ-5D) will be collected repeatedly in the patient questionnaires. As a sensitivity analysis, we will use both a general population value set from the UK,[43] as there is no such value set validated for Sweden, and a Swedish experience-based value set[38] for the translation of EQ-5D results into utilities. Sensitivity analyses will also address length of follow-up. Missing data from the questionnaires will be considered as missing at random and thus handled using a multiple imputation strategy.

Dependence of personal characteristics (e.g., education and income, collected from the LISA database at Statistics Sweden and through patient questionnaires) will be tested. The distribution of costs will also be analysed by major stakeholders, i.e. county councils/regions, market sectors (productivity loss) and individuals/families/friends to facilitate the more limited approaches to economic evaluation. For details, see Figure 1.

Design of the process evaluation

A mixed method approach will be applied as it is suitable to deal with the complexity of the intervention by combining the strength of qualitative and quantitative methodologies [46 47]. Quantitative and qualitative strands will be integrated by adding findings from different sub-projects to understand intervention outcomes and by combining methodologies within sub-

For peer review only - http://bmjopen.bmj.com/site/about/guidelines.xhtml

projects. The primary objective of the process-evaluation is to widen and deepen the understanding of intervention mechanisms of impact. Two key questions will guide the evaluation:

- How do people diagnosed with CHF and/or COPD experience that the intervention and their internal and external capabilities influenced intervention outcomes?

- How are HCPs, other invited partners and people diagnosed with CHF and/ or COPD interacting the intervention in their everyday life?

1: Exploring experiences of the intervention, internal and external capabilities in relation to program outcome

A grounded theory approach[48] will guide data collection and analysis. The method is suitable to study processes and actions and to deepen the understanding of contextual influences. Data will be collected post intervention (6-month follow-up) through individual interviews, face-to-face or by phone according to the participant's preferences. Purposeful selection of participants will be used. Initial sampling criteria are set up to reach heterogeneity regarding age, gender, diagnosis, educational level and degree of use of the intervention. 10-15 interviews are predicted to be needed but inclusion will continue until saturation is reached. A question-guide with one opening question and question areas concerning features of intervention content and design and internal/external prerequisites will be used. Interviews will be audio recorded and transcribed verbatim. Initial and focused coding will be used during analysis that is conducted by an experienced qualitative researcher. In, the initial coding, each line will be coded close to the data. Later segments of data will be synthesised and explained using a conceptual code (focused coding). Codes within and between

For peer review only

200226

interviews will be compared and sorted into categories. Memos will be used to systematically compare codes and to document analytical thoughts .[48]

2: Interactions with intervention in everyday life

A convergent parallel mixed-method design[49]will be used. User data including which features have been used from the digital platform and ratings of experienced benefits and usability will be collected at the 6-month follow-up for all participants randomised to the intervention group. These follow-ups will also include open-ended questions connected to the ratings. Self-reported use of time will also be collected for participants and HCPs. In addition, participants in the intervention group will be interviewed individually about experiences of using the intervention. For the participant interviews, purposeful sampling will be applied, sampling criteria set to reach heterogeneity and 10-15 interviews are predicted to be needed. HCPs will be interviewed using focus group methodology to obtain a collective understanding[50] followed by individual in-depth interviews. Focus groups and interviews will be conducted by an experienced researcher. All interviews will be recorded and transcribed verbatim. Descriptive statistics will be applied for quantitative data. Interviews, and answers on open ended question will be analyzed by qualitative content analysis.[51] Qualitative and quantitative data will be analysed separately, thereafter data will be merged into a combined analysis by comparing and matching patterns found in each data source.[49] For details, see Figure 1.

Ethical considerations

The study, including the consent forms for participants, was approved by the Regional Ethics Board in Gothenburg (Dnr 063-17 and T6013-18). This study conforms with the declaration

For peer review only - http://bmjopen.bmj.com/site/about/guidelines.xhtml

200226

of Helsinki.[52] All participants received oral and written informed consent before inclusion in the study.

The consent forms used in this study included information to the participants that their data would be treated confidentially and that the results from the study would be published in scientific journals such that individual persons could not be identified.

Discussion

For peer review only

This study protocol presents an RCT evaluating the effects of PCC by a combined digital platform and structured telephone support for people with COPD and/or CHF. Previous research has shown that after person-centred telephone support for people with COPD and/or CHF, the level of self-efficacy in the intervention group remained while the control group worsened .[30] However, knowledge about how to promote health in patients with COPD or CHF is limited so research about alternative ways of promoting health is warranted. This study is unique in an e-health context because it is based on PCC, which is an approach that uses patient resources and goals in the planning of care.[7 9] When providing an e-health intervention for older people (≥65 years), one plausible concern is that they might have problems in understanding and operating a digital platform. To eliminate this potential issue the HCPs will provide older patients with easy-to-follow instructions developed by a group of specialists in the areas of person-centredness, communication and pedagogics on how to create and manage their account. The participants can also contact the HCPs if they need help in managing the account.

In addition, the project intends to evaluate the intervention and identify facilitators and barriers during the process. Complex interventions, such as the one described in this protocol, have multiple interacting components. Consequently, the methods and

For peer review only - http://bmjopen.bmj.com/site/about/guidelines.xhtml

200226

procedures in such interventions are often less standardised [53] To integrate a process evaluation is therefore highly recommended.[47] A process evaluation provides the opportunity to monitor the intervention and supply information on which parts of the intervention are important and what contextual factors affect the intervention. Evaluation of user experience of the intervention aspires to provide novel knowledge that can be used in clinical practice through an amplified understanding of how the intervention is used and when and for whom it is considered acceptable and beneficial. This novel knowledge can also help to explain programme outcomes.

Interventions must also be cost-effective to be implemented. Accordingly, it is vital to integrate health economic evaluations in the design of a complex intervention. One critique that has been raised against telephone-based interventions is that they may be time-consuming and expensive. However, such interventions have been evaluated and proven cost-effective.[6 54] One example is from our previous study that evaluated PCC using a telephone support programme in which the average time of use was less than 90 minutes per patient during the 6-month study period.[30]

Conducting an intervention with different or more than one diagnosis can cause difficulties in interpreting the findings. However, this is the reality of today's healthcare system. Many older patients have more than one diagnosis and their care situation is often complex.[55] COPD and CHF are both common conditions in older people and they share several symptoms (e.g., fatigue and breathlessness) and often have similar care issues.[56] In addition, comorbidity is common in COPD and CHF.[57] Thus, including patients with both diagnoses is appropriate when reflecting on the challenges and issues facing the healthcare system today.[30]

For peer review only - http://bmjopen.bmj.com/site/about/guidelines.xhtml

200226

In open trials, there is always the risk of selection bias. To prevent this there were strict inclusion and exclusion criteria and all reasons for exclusions of potential participants were recorded.

In conclusion, this RCT, which assesses the effects of PCC by an intervention that combines a digital platform with a structured telephone support programme in patients with COPD and/or CHF, will provide clinically relevant information on the effects of PCC on self-reported symptoms, HRQoL and health economics. Our study will also offer information about the barriers and solutions when applying a digital platform together with structured telephone support based on PCC in patients with COPD or CHF.

Declarations

Authors' affiliations

1 Institute of Health and Care Sciences, Sahlgrenska Academy, University of Gothenburg, Gothenburg, Sweden.

2 Centre for Person-Centred Care (GPCC), University of Gothenburg, Gothenburg, Sweden.

3 Psychiatric Department, Sahlgrenska University Hospital, Gothenburg, Sweden.

4 Department of Molecular and Clinical Medicine, Sahlgrenska Academy, University of Gothenburg, Gothenburg, Sweden.

5 National Heart and Lung Institute, Imperial College, London, United Kingdom.

6 Närhälsan Research and Development Primary Health Care, Region Västra Götaland, Sweden.

Acknowledgements

For peer review only

For peer review only - http://bmjopen.bmj.com/site/about/guidelines.xhtml

We want to thank all the participants for sharing their experiences and the participating primary care centres for support and assistance in conducting the study. We are also grateful to patient research partner Eva Fredholm for constructive discussions throughout the research process.

Authors' contributions

All authors were involved in the design of the study. LA and SW drafted the manuscript with critical input from EB, AF, HG, EF, KS and IE. IE is the grant holder and project leader. All authors reviewed, edited and approved the final version of the manuscript.

Funding

This work was supported by The Swedish Heart & Lung Foundation (DNr.20180183), the Swedish Research Council (DNr 2017-01230) and the Centre for Person-Centred Care at the University of Gothenburg (GPCC), Sweden. The funders had no role in analysis or preparation of the manuscript.

Competing interests

The authors declare that they have no competing interests.

Patient consent for publication

Because we do not provide personal information in our manuscript, "Consent for publication" is not applicable here.

Data availability statement

The data sets generated and analysed during the current study are not publicly available for ethical reasons but are available from the corresponding author on reasonable request.

Open access

For peer review only

200226

484 This is an open access article distributed in accordance with the Creative Commons
485 Attribution Non Commercial (CC BY-NC 4.0) license, which permits others to contribute,
486 remix, adapt, build upon this work non-commercially, and license their derivative works on
487 different terms, provided the original work is properly cited, appropriate credit is given, any
488 changes made indicated and the use is non-commercial (see
489 http://creativecommons.org/licenses/by-nc/4.0/).

490 Word count

491 4433

492

For peer review only

200226

References

1. Rabe KF, Watz H. Chronic obstructive pulmonary disease. *Lancet (London, England)* 2017;389(10082):1931-40. doi: 10.1016/s0140-6736(17)31222-9 [published Online First: 2017/05/18]

2. Ponikowski P, Voors AA, Anker SD, et al. 2016 ESC Guidelines for the Diagnosis and Treatment of Acute and Chronic Heart Failure. *Revista espanola de cardiologia (English ed)* 2016;69(12):1167. doi: 10.1016/j.rec.2016.11.005 [published Online First: 2016/11/30]

3. Theander K, Hasselgren M, Luhr K, et al. Symptoms and impact of symptoms on function and health in patients with chronic obstructive pulmonary disease and chronic heart failure in primary health care. *International journal of chronic obstructive pulmonary disease* 2014;9:785.

4. Holland R, Battersby J, Harvey I, et al. Systematic review of multidisciplinary interventions in heart failure. *Heart* 2005;91(7):899-906.

5. Vogelmeier CF, Criner GJ, Martinez FJ, et al. Global strategy for the diagnosis, management, and prevention of chronic obstructive lung disease 2017 report. GOLD executive summary. *American journal of respiratory and critical care medicine* 2017;195(5):557-82.

6. Wildevuur SE, Simonse LW. Information and communication technology–enabled person-centered care for the “big five” chronic conditions: scoping review. *Journal of medical Internet research* 2015;17(3):e77.

7. Ekman I, Swedberg K, Taft C, et al. Person-centered care—Ready for prime time. *European journal of cardiovascular nursing* 2011;10(4):248-51.

8. Ahmad N, Ellins J, Krelle H, et al. Person-centred care: from ideas to action: Health Foundation 2014.

9. Sen A. Capability and well-being73. *The quality of life* 1993;30

10. Ekman I, Wolf A, Olsson L-E, et al. Effects of person-centred care in patients with chronic heart failure: the PCC-HF study. *European heart journal* 2012;33(9):1112-19.

11. Brännström M, Boman K. Effects of person-centred and integrated chronic heart failure and palliative home care. PREFER: a randomized controlled study. *European journal of heart failure* 2014;16(10):1142-51.

12. Fors A, Ekman I, Taft C, et al. Person-centred care after acute coronary syndrome, from hospital to primary care—a randomised controlled trial. *International journal of cardiology* 2015;187:693-99.

13. Hansson E, Ekman I, Swedberg K, et al. Person-centred care for patients with chronic heart failure - a cost-utility analysis. *European journal of cardiovascular nursing : journal of the Working Group on Cardiovascular Nursing of the European Society of Cardiology* 2016;15(4):276-84. doi: 10.1177/1474515114567035 [published Online First: 2015/01/18]

14. Sahlen KG, Boman K, Brannstrom M. A cost-effectiveness study of person-centered integrated heart failure and palliative home care: Based on a randomized controlled trial. *Palliative medicine* 2016;30(3):296-302. doi: 10.1177/0269216315618544 [published Online First: 2015/11/26]

15. Feldthusen C, Dean E, Forsblad-d'Elia H, et al. Effects of Person-Centered Physical Therapy on Fatigue-Related Variables in Persons With Rheumatoid Arthritis: A Randomized Controlled Trial. *Archives of physical medicine and rehabilitation* 2016;97(1):26-36. doi: 10.1016/j.apmr.2015.09.022 [published Online First: 2015/10/21]

16. Larsson A, Palstam A, Lofgren M, et al. Resistance exercise improves muscle strength, health status and pain intensity in fibromyalgia--a randomized controlled trial. *Arthritis research & therapy* 2015;17:161. doi: 10.1186/s13075-015-0679-1 [published Online First: 2015/06/19]

For peer review only - http://bmjopen.bmj.com/site/about/guidelines.xhtml

200226

17. Fors A, Taft C, Ulin K, et al. Person-centred care improves self-efficacy to control symptoms after acute coronary syndrome: a randomized controlled trial. *European Journal of Cardiovascular Nursing* 2016;15(2):186-94.
18. Wolf A, Vella R, Fors A. The impact of person-centred care on patients' care experiences in relation to educational level after acute coronary syndrome: secondary outcome analysis of a randomised controlled trial. *European journal of cardiovascular nursing : journal of the Working Group on Cardiovascular Nursing of the European Society of Cardiology* 2019;18(4):299-308. doi: 10.1177/1474515118821242 [published Online First: 2019/01/18]
19. Fors A, Gyllensten H, Swedberg K, et al. Effectiveness of person-centred care after acute coronary syndrome in relation to educational level: Subgroup analysis of a two-armed randomised controlled trial. *International journal of cardiology* 2016;221:957-62.
20. Fors A, Swedberg K, Ulin K, et al. Effects of person-centred care after an event of acute coronary syndrome: Two-year follow-up of a randomised controlled trial. *Int J Cardiol* 2017;249:42-47. doi: 10.1016/j.ijcard.2017.08.069 [published Online First: 2017/09/13]
21. Ekman I, Hedman H, Swedberg K, et al. Commentary: Swedish initiative on person centred care. *BMJ (Clinical research ed)* 2015;350:h160. doi: 10.1136/bmj.h160 [published Online First: 2015/02/12]
22. Jansson I, Fors A, Ekman I, et al. Documentation of person-centred health plans for patients with acute coronary syndrome. *European journal of cardiovascular nursing : journal of the Working Group on Cardiovascular Nursing of the European Society of Cardiology* 2018;17(2):114-22. doi: 10.1177/1474515117718415 [published Online First: 2017/06/29]
23. Sanders EB-N, Stappers PJ. Co-creation and the new landscapes of design. *Co-design* 2008;4(1):5-18.
24. Foundation TSI. The swedes and the internet 2019.: Internet Stiftelsen, 2019.
25. Erdelez S, Tanacković SF, Balog KP. Online behavior of the alzheimer's disease patient caregivers on croatian online discussion forum. *Proceedings of the Association for Information Science and Technology* 2019;56(1):78-88.
26. Ali L, Fors A, Ekman I. Need of support in people with chronic obstructive pulmonary disease. *Journal of clinical nursing* 2017
27. Frederix I, Caiani EG, Dendale P, et al. ESC e-Cardiology Working Group Position Paper: Overcoming challenges in digital health implementation in cardiovascular medicine. *European journal of preventive cardiology* 2019;26(11):1166-77.
28. Guo X, Sun Y, Wang N, et al. The dark side of elderly acceptance of preventive mobile health services in China. *Electronic Markets* 2013;23(1):49-61.
29. Hanlon P, Daines L, Campbell C, et al. Telehealth Interventions to Support Self-Management of Long-Term Conditions: A Systematic Metareview of Diabetes, Heart Failure, Asthma, Chronic Obstructive Pulmonary Disease, and Cancer. *J Med Internet Res* 2017;19(5):e172. doi: 10.2196/jmir.6688 [published Online First: 2017/05/21]
30. Fors A, Blanck E, Ali L, et al. Effects of a person-centred telephone-support in patients with chronic obstructive pulmonary disease and/or chronic heart failure–A randomized controlled trial. *PloS one* 2018;13(8):e0203031.
31. Wolf A, Fors A, Ulin K, et al. An eHealth Diary and Symptom-Tracking Tool Combined With Person-Centered Care for Improving Self-Efficacy After a Diagnosis of Acute Coronary Syndrome: A Substudy of a Randomized Controlled Trial. *J Med Internet Res* 2016;18(2):e40. doi: 10.2196/jmir.4890 [published Online First: 2016/02/26]
32. Chan AW, Tetzlaff JM, Altman DG, et al. SPIRIT 2013 Statement: defining standard protocol items for clinical trials. *Revista panamericana de salud publica = Pan American journal of public health* 2015;38(6):506-14. [published Online First: 2016/07/22]
33. Chan AW, Tetzlaff JM, Gotzsche PC, et al. SPIRIT 2013 explanation and elaboration: guidance for protocols of clinical trials. *BMJ (Clinical research ed)* 2013;346:e7586. doi: 10.1136/bmj.e7586 [published Online First: 2013/01/11]

For peer review only - http://bmjopen.bmj.com/site/about/guidelines.xhtml

200226

34. Spinuzzi C. The methodology of participatory design. *Technical Communication* 2005;52(2):163-74.
35. Bandura A. Self-efficacy: toward a unifying theory of behavioral change. *Psychological review* 1977;84(2):191.
36. Lorig KR, Ritter P, Stewart AL, et al. Chronic disease self-management program: 2-year health status and health care utilization outcomes. *Medical care* 2001;39(11):1217-23. doi: 10.1097/00005650-200111000-00008 [published Online First: 2001/10/19]
37. Schwarzer R, Jerusalem M. Generalized self-efficacy scale. *Measures in health psychology: A user's portfolio Causal and control beliefs* 1995;1(1):35-37.
38. Burstrom K, Sun S, Gerdtham UG, et al. Swedish experience-based value sets for EQ-5D health states. *Quality of life research : an international journal of quality of life aspects of treatment, care and rehabilitation* 2014;23(2):431-42. doi: 10.1007/s11136-013-0496-4 [published Online First: 2013/08/27]
39. Zigmond AS, Snaith RP. The hospital anxiety and depression scale. *Acta psychiatrica scandinavica* 1983;67(6):361-70.
40. Ekman I, Granger B, Swedberg K, et al. Measuring shortness of breath in heart failure (SOB-HF): development and validation of a new dyspnoea assessment tool. *European journal of heart failure* 2011;13(8):838-45.
41. Jones P, Brusselle G, Dal Negro R, et al. Properties of the COPD assessment test in a cross-sectional European study. *European Respiratory Journal* 2011;38(1):29-35.
42. Stenton C. The MRC breathlessness scale. *Occupational Medicine* 2008;58(3):226-27.
43. Dolan P, Gudex C, Kind P, et al. A social tariff for EuroQol: results from a UK general population survey, 1995.
44. Segel JE. Cost-of-illness studies—a primer. *RTI-UNC Center of Excellence in Health Promotion Economics* 2006:1-39.
45. Drummond MF, Drummond MF, McGuire A. Economic evaluation in health care: merging theory with practice: OUP Oxford 2001.
46. Craig P, Dieppe P, Macintyre S, et al. Developing and evaluating complex interventions: the new Medical Research Council guidance. *BMJ (Clinical research ed)* 2008;337:a1655. doi: 10.1136/bmj.a1655 [published Online First: 2008/10/01]
47. Moore GF, Audrey S, Barker M, et al. Process evaluation of complex interventions: Medical Research Council guidance. *BMJ (Clinical research ed)* 2015;350:h1258.
48. Charmaz K. Constructing grounded theory: sage 2014.
49. Fetters MD, Curry LA, Creswell JW. Achieving integration in mixed methods designs—principles and practices. *Health services research* 2013;48(6pt2):2134-56.
50. Krueger RA. Focus groups: A practical guide for applied research: Sage publications 2014.
51. Graneheim UH, Lundman B. Qualitative content analysis in nursing research: concepts, procedures and measures to achieve trustworthiness. *Nurse education today* 2004;24(2):105-12.
52. World Medical Association Declaration of Helsinki: ethical principles for medical research involving human subjects. *Jama* 2013;310(20):2191-4. doi: 10.1001/jama.2013.281053 [published Online First: 2013/10/22]
53. Campbell NC, Murray E, Darbyshire J, et al. Designing and evaluating complex interventions to improve health care. *BMJ (Clinical research ed)* 2007;334(7591):455-59.
54. Inglis SC, Clark RA, Dierckx R, et al. Structured telephone support or non-invasive telemonitoring for patients with heart failure. *Cochrane Database of Systematic Reviews* 2015(10)
55. Alfredsson J, Alexander KP. Multiple chronic conditions in older adults with acute coronary syndromes. *Clinics in geriatric medicine* 2016;32(2):291-303.
56. Dharmarajan K, Strait KM, Tinetti ME, et al. Treatment for multiple acute cardiopulmonary conditions in older adults hospitalized with pneumonia, chronic obstructive pulmonary disease, or heart failure. *Journal of the American Geriatrics Society* 2016;64(8):1574-82.

For peer review only

For peer review only - http://bmjopen.bmj.com/site/about/guidelines.xhtml

200226

57. Hawkins NM, Petrie MC, Jhund PS, et al. Heart failure and chronic obstructive pulmonary disease: diagnostic pitfalls and epidemiology. *Eur J Heart Fail* 2009;11(2):130-9. doi: 10.1093/eurjhf/hfn013 [published Online First: 2009/01/27]

For peer review only

For peer review only - http://bmjopen.bmj.com/site/about/guidelines.xhtml

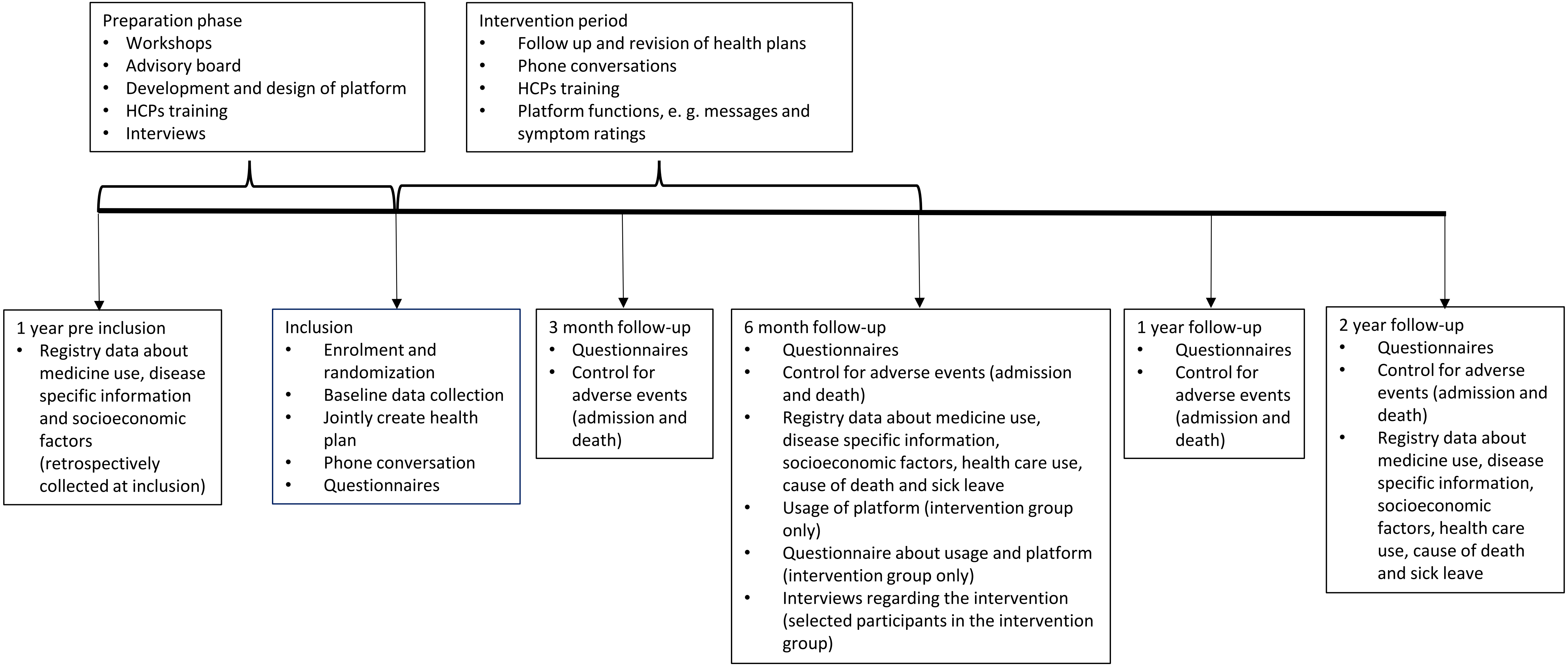
Preparation phase
• Workshops
• Advisory board
• Development and design of platform
• HCPs training
• Interviews
Intervention period
• Follow up and revision of health plans
• Phone conversations
• HCPs training
• Platform functions, e. g. messages and symptom ratings
1 year pre inclusion
• Registry data about medicine use, disease specific information and socioeconomic factors (retrospectively collected at inclusion)
Inclusion
• Enrolment and randomization
• Baseline data collection
• Jointly create health plan
• Phone conversation
• Questionnaires
3 month follow-up
• Questionnaires
• Control for adverse events (admission and death)
6 month follow-up
• Questionnaires
• Control for adverse events (admission and death)
• Registry data about medicine use, disease specific information, socioeconomic factors, health care use, cause of death and sick leave
• Usage of platform (intervention group only)
• Questionnaire about usage and platform (intervention group only)
• Interviews regarding the intervention (selected participants in the intervention group)
1 year follow-up
• Questionnaires
• Control for adverse events (admission and death)
2 year follow-up
• Questionnaires
• Control for adverse events (admission and death)
• Registry data about medicine use, disease specific information, socioeconomic factors, health care use, cause of death and sick leave

GÖTEBORGS UNIVERSITET

FORSKNINGSPERSONSINFORMATION

Vi vill tillfråga dig om att delta i en studie som utvärderar personcentrerad vård på distans

Studien heter:
PROTECT - Personcentrerad vård på distans via ett e-Hälsa stöd för personer med kronisk hjärtsvikt och/eller obstruktiv lungsjukdom.

Bakgrund och syfte
Personcentrerad vård bygger på ett partnerskap mellan professionella inom hälso- och sjukvård och patient. I praktiken betyder detta att personal (ofta läkare eller sjuksköterska) tillsammans med patienten (ibland tillsammans med närstående) formulerar en personlig hälsoplan. Personcentrerad vård har utvärderats i flera studier och goda effekter i form av till exempel högre livskvalitet och tryggare patienter har visats. Vi vill nu utveckla och testa om det går att ha ett personcentrerat arbetssätt på distans (via internet) för patienter med kronisk hjärtsvikt och/eller kronisk obstruktiv lungsjukdom (KOL).

Förfrågan om deltagande
Ditt deltagande är frivilligt. Du har, när som helst, utan särskild förklaring, rätt att avbryta fortsatt deltagande. Det kommer inte att påverka din övriga vård eller behandling.

Hur går studien till?
Vi jämför personcentrerad vård som tillägg till sedvanlig vård med enbart sedvanlig vård. Detta görs genom att deltagare lottas till antingen interventions- eller kontrollgruppen. Du som lottas till interventionsgruppen, det vill säga ett personcentrerat arbetssätt på distans kommer att kontaktas via telefon av en sjuksköterska och en personlig hälsoplan görs. Dessa samtal kan komma att spelas in, men du tillfrågas i så fall om detta och har naturligtvis rätt att avböja.

Fortsatt kontakt med sjuksköterskan sker via internet (dator, I-pad, smart-phone) och du och sjuksköterskan kan både ringa och skicka meddelande till varandra. På den digitala plattformen kan du se din hälsoplan och tillsammans med sjuksköterskan diskutera och följa upp hälsoplanen. Du kan också rapportera hur du mår, skriva hälsodagbok och bjuda in vänner och anhöriga till den digitala plattformen. Informationen i din hälsoplan är din egen och du bestämmer själv vem du vill visa upp den för. Din användning och dina rapporteringar och dagboksanteckningar på den digitala plattformen kan komma att registreras, i syfte att få en bild av hur plattformen används. Inom ramen för forskningsstudien kommer alla hälsoplaner att analyseras av forskare knutna till studien. Du kommer eventuellt också att tillfrågas om att bli intervjuad och den intervjun kan komma att spelas in. All information kommer att behandlas konfidentiellt enligt de

riktlinjer som finns vid Göteborgs Universitet, var god se avsnitt om hantering av data längre ner i dokumentet.

Du som kommer med i kontrollgruppen kommer att få vård såsom tidigare, alltså inte tillgång till den digitala plattformen.

Deltagare i båda grupperna kommer att besvara ett antal enkäter (tidsåtgång cirka 20-30 minuter), dessa berör upplevelse av symtom, livskvalitet och självtillit. Enkäterna kommer att besvaras vid fem tillfällen. Första gången efter att du inkluderats i studien, sedan efter 3, 6, 12 och slutligen efter 24 månader,

Oavsett vilken grupp du lottas till kommer din medicinska behandling att fortsätta enligt de ordinationer du har sedan tidigare.

Vilka är riskerna?
Personcentrerad vård innebär ett delat ansvar mellan professionella inom hälso- och sjukvård och patient. Om du lottas till gruppen som får tillgång till den digitala plattformen kan det innebära att din roll som patient och vårdgivarrollen blir nedtonad och en vänskaps- snarare än professionell relation kan utvecklas. Att svara på frågor i en enkät eller under en intervju innebär ingen fysisk smärta eller obehag. En del av frågorna i enkäterna kan upplevas som personliga och möjligen även väcka en del känsliga tankar. Om du inte vill fortsätta att besvara frågorna eller avsluta en eventuell intervju kan du när som helst avbryta utan att ange skäl.

Finns det några fördelar?
En möjlig nytta är att de som lottas till att få tillgång till den digitala plattformen kan uppleva ett bättre stöd under tiden som interventionen pågår. På sikt är tanken att båda grupperna bidrar till att bland annat få kunskap om vilka faktorer som stärker patienters självtillit och minskar risken för att läggas in på sjukhus, resultat som bidrar till att kunna utveckla och förbättra vården.

Hantering av data och sekretess
Om du väljer att delta innebär det att vi kommer att använda uppgifter från din patientjournal och även begära uppgifter från olika register från det datum du inkluderas i studien till ditt slutdatum i studien (24 månader efter inklusion). De register som omfattas är; det nationella registret för hjärt- och kärlsjukdom (SWEDEHEART RIKS-HIA) respektive luftvägsregistret (Riks-KOL), uppgifter om inkomst och sysselsättning från Statistiska Centralbyråns register (Lisa-databasen), uppgifter om vårdkonsumtion från Västra Götalandsregionens VEGA-databas, från Socialstyrelsen hämtas uppgifter om förskrivna läkemedel och relaterade kostnader från läkemedelsregistret och eventuella uppgifter från dödsorsaksregistret, samt uppgifter om sjukskrivning och sjukpensionering från Försäkringskassans register MiDAS. Du har rätt att begära tillgång till personuppgifterna och få dessa rättade eller raderade samt att få behandlingen av uppgifterna begränsad. Data från studien kommer att lagras och databehandlas. All data kommer att kodas och behandlas så att inte obehöriga kan ta del av dem. Data kommer att förvaras på ett säkert sätt, i ett låst och brandsäkert utrymme. Kodlistor förvaras åtskilt från data och data sparas i 10 år för att möjliggöra granskning. Uppgifterna är sekretesskyddade. Informationen som vi samlar in kommer att analyseras på Göteborgs universitet och kommer inte kunna knytas till dig som person. Alla uppgifter kommer att behandlas enligt

For peer review only - http://bmjopen.bmj.com/site/about/guidelines.xhtml

dataskyddsförordningen (GDPR). Ansvariga för dina personuppgifter är Göteborgs universitet och dataskyddsombud är Kristina Ullgren (Telefon 031-7861092). Om du är missnöjd med hur dina personuppgifter behandlas har du rätt att lämna in klagomål till Datainspektionen, som är tillsynsmyndighet. Resultaten från studien kommer att publiceras i en vetenskaplig tidskrift, enskilda individer kommer inte att kunna identifieras. Om Du har frågor kan Du vända Dig till studieansvarig.

Hur får jag information om studiens resultat?

Om du hör av dig till ansvariga för studien (v.g. se nedan) så skickar vi publicerat material till dig.

Ansvariga för studien
Inger Ekman, leg. sjuksköterska och professor
Tel: 031-786 6014, 0739-81 57 65

Karl Swedberg, överläkare och professor
Tel: 0707-144747

Kontaktpersoner:
Mahboubeh Goudarzi, leg. sjuksköterska, forskningsassistent tel.: 031-343 5923
Emma Vasell, leg. sjuksköterska, forskningsassistent tel.: 031-343 5378
Mobil nummer: 0700-207953

Reporting checklist for protocol of a clinical trial.

Based on the SPIRIT guidelines.

Instructions to authors

Complete this checklist by entering the page numbers from your manuscript where readers will find each of the items listed below.

Your article may not currently address all the items on the checklist. Please modify your text to include the missing information. If you are certain that an item does not apply, please write "n/a" and provide a short explanation.

Upload your completed checklist as an extra file when you submit to a journal.

In your methods section, say that you used the SPIRITreporting guidelines, and cite them as:

Chan A-W, Tetzlaff JM, Altman DG, Laupacis A, Gøtzsche PC, Krleža-Jerić K, Hróbjartsson A, Mann H, Dickersin K, Berlin J, Doré C, Parulekar W, Summerskill W, Groves T, Schulz K, Sox H, Rockhold FW, Rennie D, Moher D. SPIRIT 2013 Statement: Defining standard protocol items for clinical trials. Ann Intern Med. 2013;158(3):200-207

		Reporting Item	Page Number
**Administrative information**			
Title	#1	Descriptive title identifying the study design, population, interventions, and, if applicable, trial acronym	1
Trial registration	#2a	Trial identifier and registry name. If not yet registered, name of intended registry	2
Trial registration: data set	#2b	All items from the World Health Organization Trial Registration Data Set	2
Protocol version	#3	Date and version identifier	Top of page
Funding	#4	Sources and types of financial, material, and other support	23
Roles and	#5a	Names, affiliations, and roles of protocol contributors	1,23

For peer review only

responsibilities: contributorship			
Roles and responsibilities: sponsor contact information	#5b	Name and contact information for the trial sponsor	23
Roles and responsibilities: sponsor and funder	#5c	Role of study sponsor and funders, if any, in study design; collection, management, analysis, and interpretation of data; writing of the report; and the decision to submit the report for publication, including whether they will have ultimate authority over any of these activities	23
Roles and responsibilities: committees	#5d	Composition, roles, and responsibilities of the coordinating centre, steering committee, endpoint adjudication committee, data management team, and other individuals or groups overseeing the trial, if applicable (see Item 21a for data monitoring committee)	N/A
**Introduction**			
Background and rationale	#6a	Description of research question and justification for undertaking the trial, including summary of relevant studies (published and unpublished) examining benefits and harms for each intervention	4-7
Background and rationale: choice of comparators	#6b	Explanation for choice of comparators	4-7
Objectives	#7	Specific objectives or hypotheses	7
Trial design	#8	Description of trial design including type of trial (eg, parallel group, crossover, factorial, single group), allocation ratio, and framework (eg, superiority, equivalence, non-inferiority, exploratory)	8
**Methods: Participants, interventions, and outcomes**			

For peer review only

Study setting	#9	Description of study settings (eg, community clinic, academic hospital) and list of countries where data will be collected. Reference to where list of study sites can be obtained	8-9
Eligibility criteria	#10	Inclusion and exclusion criteria for participants. If applicable, eligibility criteria for study centres and individuals who will perform the interventions (eg, surgeons, psychotherapists)	9
Interventions: description	#11a	Interventions for each group with sufficient detail to allow replication, including how and when they will be administered	10-13
Interventions: modifications	#11b	Criteria for discontinuing or modifying allocated interventions for a given trial participant (eg, drug dose change in response to harms, participant request, or improving / worsening disease)	10-13
Interventions: adherance	#11c	Strategies to improve adherence to intervention protocols, and any procedures for monitoring adherence (eg, drug tablet return; laboratory tests)	N/A
Interventions: concomitant care	#11d	Relevant concomitant care and interventions that are permitted or prohibited during the trial	N/A
Outcomes	#12	Primary, secondary, and other outcomes, including the specific measurement variable (eg, systolic blood pressure), analysis metric (eg, change from baseline, final value, time to event), method of aggregation (eg, median, proportion), and time point for each outcome. Explanation of the clinical relevance of chosen efficacy and harm outcomes is strongly recommended	13-14
Participant timeline	#13	Time schedule of enrolment, interventions (including any run-ins and washouts), assessments, and visits for participants. A schematic diagram is highly recommended (see Figure)	15 + figure
Sample size	#14	Estimated number of participants needed to achieve study objectives and how it was determined, including clinical and statistical assumptions supporting any sample size calculations	15

For peer review only - http://bmjopen.bmj.com/site/about/guidelines.xhtml

Recruitment	#15	Strategies for achieving adequate participant enrolment to reach target sample size	9-10
**Methods: Assignment of interventions (for controlled trials)**			
Allocation: sequence generation	#16a	Method of generating the allocation sequence (eg, computer-generated random numbers), and list of any factors for stratification. To reduce predictability of a random sequence, details of any planned restriction (eg, blocking) should be provided in a separate document that is unavailable to those who enrol participants or assign interventions	9-10
Allocation concealment mechanism	#16b	Mechanism of implementing the allocation sequence (eg, central telephone; sequentially numbered, opaque, sealed envelopes), describing any steps to conceal the sequence until interventions are assigned	9-10
Allocation: implementation	#16c	Who will generate the allocation sequence, who will enrol participants, and who will assign participants to interventions	9-10
Blinding (masking)	#17a	Who will be blinded after assignment to interventions (eg, trial participants, care providers, outcome assessors, data analysts), and how	9-10
Blinding (masking): emergency unblinding	#17b	If blinded, circumstances under which unblinding is permissible, and procedure for revealing a participant's allocated intervention during the trial	N/A
**Methods: Data collection, management, and analysis**			
Data collection plan	#18a	Plans for assessment and collection of outcome, baseline, and other trial data, including any related processes to promote data quality (eg, duplicate measurements, training of assessors) and a description of study instruments (eg, questionnaires, laboratory tests)	13-14

For peer review only

		along with their reliability and validity, if known. Reference to where data collection forms can be found, if not in the protocol	
Data collection plan: retention	#18b	Plans to promote participant retention and complete follow-up, including list of any outcome data to be collected for participants who discontinue or deviate from intervention protocols	13-14
Data management	#19	Plans for data entry, coding, security, and storage, including any related processes to promote data quality (eg, double data entry; range checks for data values). Reference to where details of data management procedures can be found, if not in the protocol	19-20
Statistics: outcomes	#20a	Statistical methods for analysing primary and secondary outcomes. Reference to where other details of the statistical analysis plan can be found, if not in the protocol	13-19
Statistics: additional analyses	#20b	Methods for any additional analyses (eg, subgroup and adjusted analyses)	13-19
Statistics: analysis population and missing data	#20c	Definition of analysis population relating to protocol non-adherence (eg, as randomised analysis), and any statistical methods to handle missing data (eg, multiple imputation)	13-19
**Methods: Monitoring**			
Data monitoring: formal committee	#21a	Composition of data monitoring committee (DMC); summary of its role and reporting structure; statement of whether it is independent from the sponsor and competing interests; and reference to where further details about its charter can be found, if not in the protocol. Alternatively, an explanation of why a DMC is not needed	N/A
Data monitoring: interim analysis	#21b	Description of any interim analyses and stopping guidelines, including who will have access to these interim results and make the final decision to terminate the trial	N/A
Harms	#22	Plans for collecting, assessing, reporting, and managing solicited and spontaneously reported adverse events and	13-19 + Figure

For peer review only

		other unintended effects of trial interventions or trial conduct	
Auditing	#23	Frequency and procedures for auditing trial conduct, if any, and whether the process will be independent from investigators and the sponsor	N/A
**Ethics and dissemination**			
Research ethics approval	#24	Plans for seeking research ethics committee / institutional review board (REC / IRB) approval	19-20
Protocol amendments	#25	Plans for communicating important protocol modifications (eg, changes to eligibility criteria, outcomes, analyses) to relevant parties (eg, investigators, REC / IRBs, trial participants, trial registries, journals, regulators)	N/A
Consent or assent	#26a	Who will obtain informed consent or assent from potential trial participants or authorised surrogates, and how (see Item 32)	N/A
Consent or assent: ancillary studies	#26b	Additional consent provisions for collection and use of participant data and biological specimens in ancillary studies, if applicable	N/A
Confidentiality	#27	How personal information about potential and enrolled participants will be collected, shared, and maintained in order to protect confidentiality before, during, and after the trial	19-20
Declaration of interests	#28	Financial and other competing interests for principal investigators for the overall trial and each study site	23
Data access	#29	Statement of who will have access to the final trial dataset, and disclosure of contractual agreements that limit such access for investigators	23
Ancillary and post trial care	#30	Provisions, if any, for ancillary and post-trial care, and for compensation to those who suffer harm from trial participation	N/A
Dissemination policy: trial results	#31a	Plans for investigators and sponsor to communicate trial results to participants, healthcare professionals, the public, and other relevant groups (eg, via publication,	19-20

For peer review only

		reporting in results databases, or other data sharing arrangements), including any publication restrictions	
Dissemination policy: authorship	#31b	Authorship eligibility guidelines and any intended use of professional writers	N/A
Dissemination policy: reproducible research	#31c	Plans, if any, for granting public access to the full protocol, participant-level dataset, and statistical code	23

Appendices

Informed consent materials	#32	Model consent form and other related documentation given to participants and authorised surrogates	Appendix
Biological specimens	#33	Plans for collection, laboratory evaluation, and storage of biological specimens for genetic or molecular analysis in the current trial and for future use in ancillary studies, if applicable	N/A

None The SPIRIT checklist is distributed under the terms of the Creative Commons Attribution License CC-BY-ND 3.0. This checklist can be completed online using https://www.goodreports.org/, a tool made by the EQUATOR Network in collaboration with Penelope.ai

For peer review only

BMJ Open

Person-centred care by a combined digital platform and structured telephone support for people with chronic obstructive pulmonary disease and/or chronic heart failure: Study protocol for the PROTECT randomised controlled trial

Journal:	*BMJ Open*
Manuscript ID	bmjopen-2019-036356.R2
Article Type:	Protocol
Date Submitted by the Author:	22-May-2020
Complete List of Authors:	Ali, Lilas; Institute of Health and Care Sciences, Sahlgrenska Academy, University of Gothenburg; University of Gothenburg Centre for Person-Centred Care, Sahlgrenska Academy, University of Gothenburg<br>Wallström, Sara; Institute of Health and Care Sciences, Sahlgrenska Academy, University of Gothenburg; University of Gothenburg Centre for Person-Centred Care, Sahlgrenska Academy, University of Gothenburg<br>Barenfeld, Emmelie; Insitute of Health and Care Sciences, Sahgrenska Academy, University of Gothenburg; University of Gothenburg Centre for Person-Centred Care, Sahlgrenska Academy<br>Fors, Andreas; Institute of Health of Care Sciences, Sahlgrenska Academy, University of Gothenburg; Gothenburg Centre for Person-Centred Care (GPCC),<br>Fredholm, Eva; The Swedish Heart & Lung Foundation<br>Gyllensten, Hanna; Health of Care Sciences, Sahlgrenska Academy, University of Gothenburg; Gothenburg Centre for Person-Centred Care (GPCC)<br>Swedberg, Karl; University of Gothenburg, Department of emergency and cardiovascular me; University of Gothenburg Centre for Person-Centred Care, Sahlgrenska Academy<br>Ekman, Inger; Institute of Health and Care Sciences, Sahlgrenska Academy, University of Gothenburg; University of Gothenburg Centre for Person-Centred Care, Sahlgrenska Academy, University of Gothenburg
**Primary Subject Heading**:	Nursing
Secondary Subject Heading:	Cardiovascular medicine, General practice / Family practice, Health economics, Nursing, Respiratory medicine
Keywords:	CARDIOLOGY, Adult cardiology < CARDIOLOGY, Heart failure < CARDIOLOGY, THORACIC MEDICINE

SCHOLARONE™
Manuscripts

I, the Submitting Author has the right to grant and does grant on behalf of all authors of the Work (as defined in the below author licence), an exclusive licence and/or a non-exclusive licence for contributions from authors who are: i) UK Crown employees; ii) where BMJ has agreed a CC-BY licence shall apply, and/or iii) in accordance with the terms applicable for US Federal Government officers or employees acting as part of their official duties; on a worldwide, perpetual, irrevocable, royalty-free basis to BMJ Publishing Group Ltd ("BMJ") its licensees and where the relevant Journal is co-owned by BMJ to the co-owners of the Journal, to publish the Work in this journal and any other BMJ products and to exploit all rights, as set out in our licence.

The Submitting Author accepts and understands that any supply made under these terms is made by BMJ to the Submitting Author unless you are acting as an employee on behalf of your employer or a postgraduate student of an affiliated institution which is paying any applicable article publishing charge ("APC") for Open Access articles. Where the Submitting Author wishes to make the Work available on an Open Access basis (and intends to pay the relevant APC), the terms of reuse of such Open Access shall be governed by a Creative Commons licence – details of these licences and which Creative Commons licence will apply to this Work are set out in our licence referred to above.

Other than as permitted in any relevant BMJ Author's Self Archiving Policies, I confirm this Work has not been accepted for publication elsewhere, is not being considered for publication elsewhere and does not duplicate material already published. I confirm all authors consent to publication of this Work and authorise the granting of this licence.

For peer review only

200522

Person-centred care by a combined digital platform and structured telephone support for people with chronic obstructive pulmonary disease and/or chronic heart failure: Study protocol for the PROTECT randomised controlled trial.

Lilas Ali[1,2,3], Sara Wallström[1,2], Emmelie Barenfeld[1,2], Andreas Fors[1,2,4], Eva Fredholm [5], Hanna Gyllensten[1,2], Karl Swedberg[1,2,6,7], Inger Ekman[1,2,7]

1 Institute of Health and Care Sciences, Sahlgrenska Academy, University of Gothenburg, Gothenburg, Sweden.

2 Centre for Person-Centred Care (GPCC), University of Gothenburg, Gothenburg, Sweden.

3 Sahlgrenska University Hospital, Psychiatric department, Gothenburg, Sweden.

4 Närhälsan Research and Development Primary Health Care, Region Västra Götaland, Sweden.

5 Patient representative, The Swedish Heart & Lung Foundation.

6 Department of Molecular and Clinical Medicine, Sahlgrenska Academy, University of Gothenburg, Gothenburg, Sweden.

7 Sahlgrenska university hospital/Östra/Department of Internal medicine and geriatrics, Gothenburg, Sweden.

Lilas Ali, Institute of Health Care Sciences, Centre for Person-Centred Care, Sahlgrenska Academy, Box 457, 405 30 Gothenburg, Sweden. lilas.ali@gu.se

For peer review only

For peer review only - http://bmjopen.bmj.com/site/about/guidelines.xhtml

200522

Abstract

Background: A core feature of chronic obstructive pulmonary disorder (COPD) and chronic heart failure (CHF) is that symptoms may change rapidly because of illness progression. Thus, these chronic conditions are associated with high rehospitalisation rates. Person-centred care (PCC) has been shown to have several benefits for patients with COPD or CHF (or both disorders) but has not yet been investigated through e-health services.

Aim: The project aims to evaluate the effects of PCC by a combined digital platform and structured telephone support for people with chronic obstructive pulmonary disease and/or chronic heart failure.

Methods and analysis: A randomised controlled trial with open, parallel groups that employs a participatory design process will be used. This project will also include process and health economic evaluations of the intervention.

Ethics and dissemination Ethical approval has been secured from the Regional Ethical Review Board in Gothenburg, Sweden (Dnr 063-17 and T063-18). Results will be presented at conferences and to health care professionals, participants and patient organisations. Findings will also be submitted for publication in peer-reviewed journals.

Trial registration: The trial was registered at ClinicalTrials.gov June 7, 2017, identifier: NCT03183817

Keywords: chronic heart failure; chronic obstructive pulmonary disease; e-health; patient-centred care; person-centred care; randomised controlled trial; telehealth.

For peer review only

For peer review only - http://bmjopen.bmj.com/site/about/guidelines.xhtml

200522

Article summary

Strengths and limitations of this study

- This project is the first RCT to evaluate the effects of PCC by a combined digital platform and structured telephone support for people with COPD and/ or CHF.
- The protocol details a complex intervention aimed at operationalising person-centred ethics.
- To answer the research questions of the study, collected data will be analysed using quantitative and qualitative methods.
- The evaluation will include patient-reported outcome measures together with health economics and process outcomes.
- Unfortunately, this study did not include individuals without a device for the Internet or those who could not read or write in Swedish.

For peer review only - http://bmjopen.bmj.com/site/about/guidelines.xhtml

200522

Introduction

Chronic heart failure (CHF) and chronic obstructive pulmonary disease (COPD), two long-term conditions common in older populations, are associated with high rehospitalisation rates and high mortality.[1 2] In addition, both conditions severely affect activities of daily living because of frequent symptoms that limit health-related quality of life (HRQoL), compelling patients to be dependent on others.[3] Patients with CHF and/or COPD frequently require hospital care and the severity of their symptoms often leads to repeated visits to hospital, outpatient or primary care.[1-3] Although pharmacological therapy has improved outcomes markedly over the past 10-15 years, management programmes are still needed to optimise care.[4] There are benefits of pulmonary rehabilitation (e.g., reduction in readmission and mortality), including exercise training, education and self-management interventions that specifically target behaviour change for patients with COPD.[5] A consistent recommendation in the treatment of CHF is management programmes designed to improve outcomes through structured follow-ups that increase patients´ self-care skills and other key issues (e.g., symptoms and disease trajectory).[2] Being affected by a chronic disease with a high symptom burden is a major challenge to living a full life. Health care professionals (HCPs), such as registered nurses, occupational therapist and physiotherapist, in conjunction with well-developed technology, can serve to enable successful self-management for individuals with severe long-term illnesses.[6]

Person-centred care

The basis of person-centred care (PCC) is to include the patient's experience, situation, capabilities and resources in the care process. PCC highlights the importance of understanding the person behind the patient as a human being with reason, will, emotions

For peer review only - http://bmjopen.bmj.com/site/about/guidelines.xhtml

200522

and needs in order for that person to be able to be an active partner in his or her care and treatment.[7] Patients (often with the help of relatives) present themselves as persons by expressing their illness narrative. This narrative includes how daily life events are being affected by the condition and treatment and creates a starting point for collaborative care and partnership between HCPs and patients that encourages and empowers patients to actively take part in finding solutions to their problems.[7 8] PCC entails not only identifying health barriers but also to confirm human capabilities and opportunities in the patients' home and local environment. PCC is based on ethical principles, inspired by the capability approach (an alternative approach to welfare economics), which also has been used in research as a theoretical frame of reference in several disciplines (e.g., in economics by the Nobel Laureate Amartya Sen).[9] All people develop their capabilities (such as a sense of responsibility for oneself and others) in relation to other people.

Previous studies evaluating PCC that for example utilizes personal health plans and symptom tracking have shown positive effects in patients with several conditions, both acute and long-term, and in different care settings. Positive outcomes include shortened hospital stays,[10] reduced re-admittance,[11] improved self-efficacy,[12] reduced health care costs,[13 14] maintained functional performance,[10] improved HRQoL,[11] improved pain management, greater stamina and enhanced self-reported health,[15] increased physical activity,[16] improved self-efficacy to manage symptoms,[17] reduced fatigue and anxiety[15] and improved experience of the care.[18] Moreover, effects have been especially prominent in patients without post-secondary education,[19] an effect that was sustained for up to 2 years.[20] These studies have applied a PCC approach in practice, wherein the needs of the

For peer review only - http://bmjopen.bmj.com/site/about/guidelines.xhtml

200522

patient are important but which also incorporates their human ability, which is an often underused resource in health care.[7 21 22]

E-health

To meet demands on accessible and efficient care one suggestion is to facilitate the partnership between HCPs and patients.[23] One such effort can be the care and support provided through e-health. Today, the Internet is frequently used as a source of information and Sweden belongs to the countries that is high up in a development of maturity of Internet use concerning the number of computers and the possibility of accessing the Internet.[24] Digital web-based care has no geographical constraints, i.e. it is potentially accessible everywhere. It also affords the possibility to reach persons that today avoids to seek help because they find it stigmatising.[25] Moreover, the possibility of having contact with HCPs in privacy, when one has the time and when questions arise, is found to be important.[26] Many people want to manage their health by themselves, especially when they are concerned about their illness. Yet people yearn to be understood and communicate with others with similar experiences.[26] E-health solutions offer these possibilities. Digital health interventions have also been shown to hold promise to improve the quality and experience of care for patients with CHF.[27]

There are, however, some concerns that need to be taken into account when designing e-health solutions. There are some indications that older people can find e-health solutions difficult to handle.[28] Previous research have also shown that e-health support, in which the users are not directly involved in the process, has low impact.[29]

For peer review only

For peer review only - http://bmjopen.bmj.com/site/about/guidelines.xhtml

200522

121 There is some research combining e-health with PCC. PCC in the form of a structured telephone support for patients with CHF and/ or COPD has been shown to mitigate worsening self-efficacy without increasing the risk of clinical events.[30] Using a digital symptom tracking tool in combination with PCC has been found to improve general self-efficacy compared with standard care.[31] E-health interventions that are developed using a participatory design and that uses personal resources are also consistent with the ethics expectations of PCC. Overall, e-health solutions are considered a promising approach to strengthen self-management among patients with CHF or COPD.[29] There is, however, a need for studies investigating the effects of PCC via e-health solutions for people with long-term illness.

Aim

The project aims to evaluate the effects of PCC by a combined digital platform and structured telephone support for people with chronic obstructive pulmonary disease and/or chronic heart failure.

Methods/ design

The design will be a randomised controlled trial (RCT) with open parallel groups. In addition, the project will include process and health economic evaluations of the study. Thus, the project will integrate the collection and analysis of both qualitative and quantitative data to answer the research questions. User experiences and process evaluations are necessary complements to each other to help understand more about enablers and barriers to self-management support using a combined digital platform and telephone support. The

For peer review only - http://bmjopen.bmj.com/site/about/guidelines.xhtml

200522

protocol complies with the SPIRIT (Standard Protocol Items: Recommendations for Interventional Trials).[32 33]

Patient and public involvement

Public involvement is a cornerstone in PCC. Therefore, this study incorporates a participatory design that assumes that all users (patients, relatives and HCPs) are involved in the study design, which has been reported to facilitate implementation.[34] Extensive consultation with members of the community took place during the development of the digital platform and design of the study. During these consultations the patient representatives requested that the platform should include functionalities such as ability for two-way communication, reliable information and advice about their condition and that it should be user friendly. The HCPs contributed with input concerning layout and technical functionalities of the platform. An advisory group consisting of patient representatives, relatives, HCPs, system-developers and researchers was formed to provide advice and co-design all of the major elements of the study design

Participants and setting

Study participants that fulfil the inclusion criteria of the study will be recruited from nine public primary care centres in Gothenburg.

Inclusion criteria

- Diagnosed with COPD or CHF
- Listed at one of the nine participating primary care centres
- Must understand written and spoken Swedish

Exclusion criteria

For peer review only

200522

- No device with Internet access, i.e. no computer, smartphone or tablet
- Severe impairment that prevents persons from using e-health support
- No registered address
- Any severe disease with an expected survival of <12 months
- Cognitive impairment
- Ongoing documented diagnosis of alcohol/drug abuse
- Other diseases that can interfere with follow-up (e.g., severe depression, other severe mental illnesses)
- Participating in another conflicting study

Enrolment and randomisation

Designated HCPs will screen medical records of listed patients at the participating primary care centres against study inclusion and exclusion criteria. Eligible patients will be sent a letter informing them about the study. The HCPs then contact the eligible patients by phone to give further information about the study and ask whether they willing to participate. If the patient agrees to participate, a consent form together with a return prepaid envelope will be sent to the patient. Once the consent form is returned to the HCPs, patients will be randomised to usual care or PCC plus usual care. The randomisation will be based on a computer-generated list created by a third party. All patients will be informed about group assignments by phone.

Usual care

Patients randomised to usual care will be managed by regular evidence-based treatment and care as outlined in treatment guidelines.[2 5]

Design of intervention

For peer review only - http://bmjopen.bmj.com/site/about/guidelines.xhtml

200522

The intervention is based on the theoretical PCC framework in which all people are seen as capable with a unique understanding of themselves and who have unique experiences, expectations, needs, preferences and resources. A central component of PCC is that the professional and patient jointly develop a personal health plan using resources identified in each patient´s illness history as well as defining potential barriers to effective care.[7] Self-efficacy as a concept is closely related to PCC as it also refers to people's belief in their capability to affect daily life events.[7 31 35] In addition, self-efficacy has been shown to have important implications for patient recovery,[36] which is related to the fact that patients confident in their ability to manage everyday life are likely to engage in rehabilitation more actively.[7 35] Steps and components of the intervention are described below.

Description of intervention elements

When the HCPs call the participants in the intervention group to inform them about the outcome of the randomisation procedure, the HCPs will also describe the intervention, assist the participants in creating a login to the platform and describe its features, and decide on a date and time for a phone call when the health care plan is created. All the dedicated HCPs participated in the development of the digital platform and received special training in performing PCC communication at a distance. Access to the digital platform will be password protected.

Telephone support and health plan

When the HCPs call the participants, they listen to their narrative about daily life events and how they are affected by the condition, what kind of problems they experience and what resources they have to co-create the health plan. The HCPs try to encourage narration and establish a partnership by using PCC communication skills such as open-ended questions,

For peer review only - http://bmjopen.bmj.com/site/about/guidelines.xhtml

200522

reflections and summaries. Based on the patient narrative, patient goals, resources and needs are identified and communicated with the patient (participant). The participant (sometimes together with relatives) and the HCPs formulate a person-centred health plan in partnership. The health plan contains five elements:

1. Today we have talked about
2. You would like to be able to do/feel
3. To get there you have to
4. Capabilities and resources that could help you are
5. Support that you might need is

The health plan will be based on mutual agreement between the patient and HCPs and contains the patient’s goals, resources and support needs. It also contains follow-up actions and steps needed to achieve progress in the care of the patient. The date of follow-up conversations will be scheduled jointly. This plan will be uploaded to the digital platform with help from the HCPs if the participants have chosen to write the plan themselves or it will be written directly by the HCPs and then finalised when accepted by the participants.

The health plan will be the point of departure for the forthcoming conversations and communication via the platform that the participants and the HCPs will have during the study period (of 6 months). The plan will be considered during each follow-up when the HCPs contact the participants and revised when necessary. During the intervention, the participants will also be free to contact the HCPs during office hours.

Digital platform

For peer review only - http://bmjopen.bmj.com/site/about/guidelines.xhtml

200522

231 The platform will contain headings such as; messages, daily ratings, health plan, contact, next of kin and links, when logged in that may inspire the patients to make notes on “a good day” respectively “a bad day”. Functionalities of the platform include ability for two-way communication, possibility to rate symptoms and a collection of links to information concerning COPD and CHF. Symptom-ratings and comments will also be made to create a graph over time once the daily ratings have been completed. The HCPs will be able to see the patients´ accounts and make comments. The patients will be able to add or delete any person (for example informal carers, family or friends) that they would like to give access to their account. The patients will also be able to customise what content they want visible for invited persons. For instance, they could choose to restrict visibility of the messages between themselves and the HCPs or elect to show all content in their account such as the health plan and daily ratings.

243 The digital platform will also contain a function to send private messages between the participants and the HCPs. The HCPs check for new messages daily. Another function offered in the platform will be the possibility to link relevant information about CHF and COPD that is provided by patient organisations (e.g., the heart and lung association) and the Swedish national help guide (1177.se). There will also be links to a peer-to-peer support group.

249 Sources and collection of data

250 Data will be collected using self-report instruments, interviews, study documentation, medical records and administrative registers. Questionnaires will be mailed (together with a prepaid return envelope) to the participants at study inclusion and 3, 6, 12 and 24 months after randomisation. Paper questionnaires were chosen since not all of the instruments are

For peer review only - http://bmjopen.bmj.com/site/about/guidelines.xhtml

validated for online use. In connection with the follow-ups and adverse events (i.e. admission to hospital or death) will be controlled for all participants. Baseline data (including demographics, characteristics and medical history) will be collected from the patients' medical records. For details, see Figure 1.

Questionnaires

- The General Self-Efficacy Scale (GSE)[37]
- EuroQol-5 Dimensions health state questionnaire (EQ-5D)[38]
- Hospital Anxiety and Depression Scale (HADS)[39]
- Shortness of Breath in Heart Failure (SOB-HF)[40]
- COPD Assessment Test (CAT)[41]
- The Medical Research Council (MRC) breathlessness scale[42]

Primary endpoint

The primary endpoint will be the composite score of change in general self-efficacy and rehospitalisation or death 6 months after randomisation.

A patient will be classified as deteriorated if:

- general self-efficacy has decreased by ≥5 units or
- patient has been hospitalised for unscheduled reasons due to COPD/ CHF or
- patient has died due to all causes.

A patient is classified as improved if:

- general self-efficacy has increased by ≥5 units and

For peer review only

200522

- the patient has not been hospitalised due to COPD/CHF and
- the patient has not died.

Those who have neither deteriorated nor improved are considered unchanged.

Secondary endpoints

a. Number of admissions

b. General self-efficacy[37]

c. Health care utilisation measured as the number of admissions and unscheduled outpatient visits due to unplanned visits to hospital or primary care centre due to symptoms of COPD or CHF

d. HADS[39]

e. HRQoL (EQ-5D)[43]

f. Incremental cost-utility ratios

g. Comparing HRQoL and costs for health care

h. CAT[41]

i. The MRC breathlessness scale[42]

j. SOB-HF[40]

Project timeline

Recruitment of participants commenced in August 2017 and was completed in June 2019. During this period, 224 eligible patients had been identified and included in the study. The

For peer review only

For peer review only - http://bmjopen.bmj.com/site/about/guidelines.xhtml

intervention will continue until early 2020 after which data for the primary outcome will be collected. Data collection for the secondary outcome measures will continue until mid-2021.

Power calculation

To achieve 80% power based on a p-value of 0.05 (two-tailed) for an increase in the proportion of improved patients from 20 to 40% in the primary outcome a sample size of 91 patients in each group is required. However, we plan to include 110 patients in each group to have some margin for withdrawals/dropouts. Thus, a minimum of 220 patients will be randomised across the two groups.

For peer review only

Quantitative data analysis

Descriptive statistics will be used to characterise the study groups. Between-group differences will be tested using Fisher's exact test for dichotomous variables and parametric and non-parametric tests will be used for continuous variables. Logistic regression will be used to calculate odds ratios with 95% confidence intervals. Statistical significance will be set to $p<0.05$ (two-sided). Both intention-to-treat and per-protocol analyses will be conducted. In addition, subgroup analyses will be performed.

Design of the health economic evaluation

The objective of the health-economic analysis is to estimate differences in total societal costs between the two approaches in relation to the difference in quality-adjusted life-years gained (incremental cost-utility ratios) over the two-year follow-up. This analysis will be followed by analysing its dependence on personal characteristics. The estimation includes two steps: a) quantify all resource use related to illness and treatment, as well as the HRQoL in physical terms and b) evaluate uses of resources in monetary terms and calculate a utility measure between minus and plus one from the HRQoL instrument[44]. A ratio will also be

For peer review only - http://bmjopen.bmj.com/site/about/guidelines.xhtml

200522

created that is the incremental cost (the cost difference between intervention and usual care groups) divided by the incremental effect (corresponding difference in utility).

Resource use will include data from the regional patient register VEGA (held by Region Västra Götaland), which covers all care reimbursed by the region, including hospital, outpatient and primary care conducted within the region but also care received in other regions. Information on prescribed medication and related costs will be obtained from the National Board of Health and Welfare. In addition, data on lost productivity for the patient due to sickness absence (i.e. absence from work that can be attributed to sickness) will be retrieved from the MiDAS database (Social Insurance Agency) and self-report in patient questionnaires (since the first 14 days of each sick-leave are not covered by official statistics). This information will be used to estimate indirect costs (sometimes called productivity costs) using the human capital approach, which entails multiplying the net days on sick leave by the mean wage and associated social security contributions. The difference in costs and utilities will be compared (assuming that there will be no difference in length of life between the two groups) and cost-utility ratios estimated. Several issues are involved in valuing health care in monetary terms related to the basic economic-theoretical concept of "opportunity cost", true societal valuations and marginal costs. We will follow the state of the art practice by making alternative estimates of costs and present the results as sensitivity analyses.[45] Such analyses will be a test of the robustness of the results. HRQoL (measured by EQ-5D) will be collected repeatedly in the patient questionnaires. As a sensitivity analysis, we will use both a general population value set from the UK,[43] as there is no such value set validated for Sweden, and a Swedish experience-based value set[38] for the translation of EQ-5D results into utilities. Sensitivity analyses will also address length of

For peer review only - http://bmjopen.bmj.com/site/about/guidelines.xhtml

200522

339 follow-up. Missing data from the questionnaires will be considered as missing at random and thus handled using a multiple imputation strategy.

341 Dependence of personal characteristics (e.g., education and income, collected from the LISA database at Statistics Sweden and through patient questionnaires) will be tested. The distribution of costs will also be analysed by major stakeholders, i.e. county councils/regions, market sectors (productivity loss) and individuals/families/friends to facilitate the more limited approaches to economic evaluation. For details, see Figure 1.

Design of the process evaluation

347 A mixed method approach will be applied as it is suitable to deal with the complexity of the intervention by combining the strength of qualitative and quantitative methodologies.[46 47] Quantitative and qualitative strands will be integrated by adding findings from different sub-projects to understand intervention outcomes and by combining methodologies within sub-projects. The primary objective of the process-evaluation is to widen and deepen the understanding of intervention mechanisms of impact. Two key questions will guide the evaluation:

- How do people diagnosed with CHF and/or COPD experience that the intervention and their internal and external capabilities influenced intervention outcomes?
- How are HCPs, other invited partners and people diagnosed with CHF and/ or COPD interacting the intervention in their everyday life?

1: Exploring experiences of the intervention, internal and external capabilities in relation to program outcome

For peer review only - http://bmjopen.bmj.com/site/about/guidelines.xhtml

200522

A grounded theory approach[48] will guide data collection and analysis. The method is suitable to study processes and actions and to deepen the understanding of contextual influences. Data will be collected post intervention (6-month follow-up) through individual interviews, face-to-face or by phone according to the participant's preferences. Purposeful selection of participants will be used. Initial sampling criteria are set up to reach heterogeneity regarding age, gender, diagnosis, educational level and degree of use of the intervention. 10-15 interviews are predicted to be needed but inclusion will continue until saturation is reached. A question-guide with one opening question and question areas concerning features of intervention content and design and internal/external prerequisites will be used. Interviews will be audio recorded and transcribed verbatim. Initial and focused coding will be used during analysis that is conducted by an experienced qualitative researcher. In, the initial coding, each line will be coded close to the data. Later segments of data will be synthesised and explained using a conceptual code (focused coding). Codes within and between interviews will be compared and sorted into categories. Memos will be used to systematically compare codes and to document analytical thoughts.[48]

2: Interactions with intervention in everyday life

A convergent parallel mixed-method design[49]will be used. User data including which features have been used from the digital platform and ratings of experienced benefits and usability will be collected at the 6-month follow-up for all participants randomised to the intervention group. These follow-ups will also include open-ended questions connected to the ratings. Self-reported use of time will also be collected for participants and HCPs. In addition, participants in the intervention group will be interviewed individually about

For peer review only - http://bmjopen.bmj.com/site/about/guidelines.xhtml

200522

382 experiences of using the intervention. For the participant interviews, purposeful sampling will be applied, sampling criteria set to reach heterogeneity and 10-15 interviews are predicted to be needed. HCPs will be interviewed using focus group methodology to obtain a collective understanding[50] followed by individual in-depth interviews. Focus groups and interviews will be conducted by an experienced researcher. All interviews will be recorded and transcribed verbatim. Descriptive statistics will be applied for quantitative data. Interviews, and answers on open ended question will be analyzed by qualitative content analysis.[51] Qualitative and quantitative data will be analysed separately, thereafter data will be merged into a combined analysis by comparing and matching patterns found in each data source.[49] For details, see Figure 1.

Ethics and dissemination

The study, including the consent forms for participants, was approved by the Regional Ethics Board in Gothenburg (Dnr 063-17 and T6013-18). This study conforms with the declaration of Helsinki.[52] All participants received oral and written informed consent before inclusion in the study.

The consent forms used in this study included information to the participants that their data would be treated confidentially and that the results from the study would be published in scientific journals such that individual persons could not be identified.

Discussion

This study protocol presents an RCT evaluating the effects of PCC by a combined digital platform and structured telephone support for people with COPD and/or CHF. Previous research has shown that after person-centred telephone support for people with COPD

For peer review only - http://bmjopen.bmj.com/site/about/guidelines.xhtml

200522

and/or CHF, the level of self-efficacy in the intervention group remained while the control group worsened.[30] However, knowledge about how to promote health in patients with COPD or CHF is limited so research about alternative ways of promoting health is warranted. This study is unique in an e-health context because it is based on PCC, which is an approach that uses patient resources and goals in the planning of care.[7 9] When providing an e-health intervention for older people (≥65 years), one plausible concern is that they might have problems in understanding and operating a digital platform. To eliminate this potential issue the HCPs will provide older patients with easy-to-follow instructions developed by a group of specialists in the areas of person-centredness, communication and pedagogics on how to create and manage their account. The participants can also contact the HCPs if they need help in managing the account.

In addition, the project intends to evaluate the intervention and identify facilitators and barriers during the process. Complex interventions, such as the one described in this protocol, have multiple interacting components. Consequently, the methods and procedures in such interventions are often less standardised.[53] To integrate a process evaluation is therefore highly recommended.[47] A process evaluation provides the opportunity to monitor the intervention and supply information on which parts of the intervention are important and what contextual factors affect the intervention. Evaluation of user experience of the intervention aspires to provide novel knowledge that can be used in clinical practice through an amplified understanding of how the intervention is used and when and for whom it is considered acceptable and beneficial. This novel knowledge can also help to explain programme outcomes.

For peer review only - http://bmjopen.bmj.com/site/about/guidelines.xhtml

Interventions must also be cost-effective to be implemented. Accordingly, it is vital to integrate health economic evaluations in the design of a complex intervention. One critique that has been raised against telephone-based interventions is that they may be time-consuming and expensive. However, such interventions have been evaluated and proven cost-effective.[6 54] One example is from our previous study that evaluated PCC using a telephone support programme in which the average time of use was less than 90 minutes per patient during the 6-month study period.[30]

Conducting an intervention with different or more than one diagnosis can cause difficulties in interpreting the findings. However, this is the reality of today's healthcare system. Many older patients have more than one diagnosis and their care situation is often complex.[55] COPD and CHF are both common conditions in older people and they share several symptoms (e.g., fatigue and breathlessness) and often have similar care issues.[56] In addition, comorbidity is common in COPD and CHF.[57] Thus, including patients with both diagnoses is appropriate when reflecting on the challenges and issues facing the healthcare system today.[30] In open trials, there is always the risk of selection bias. To prevent this there were strict inclusion and exclusion criteria and all reasons for exclusions of potential participants were recorded.

In conclusion, this RCT, which assesses the effects of PCC by an intervention that combines a digital platform with a structured telephone support programme in patients with COPD and/or CHF, will provide clinically relevant information on the effects of PCC on self-reported symptoms, HRQoL and health economics. Our study will also offer information

For peer review only - http://bmjopen.bmj.com/site/about/guidelines.xhtml

200522

about the barriers and solutions when applying a digital platform together with structured telephone support based on PCC in patients with COPD or CHF.

Declarations

Authors' affiliations

1 Institute of Health and Care Sciences, Sahlgrenska Academy, University of Gothenburg, Gothenburg, Sweden.

2 Centre for Person-Centred Care (GPCC), University of Gothenburg, Gothenburg, Sweden.

3 Psychiatric Department, Sahlgrenska University Hospital, Gothenburg, Sweden.

4 Department of Molecular and Clinical Medicine, Sahlgrenska Academy, University of Gothenburg, Gothenburg, Sweden.

5 National Heart and Lung Institute, Imperial College, London, United Kingdom.

6 Närhälsan Research and Development Primary Health Care, Region Västra Götaland, Sweden.

Acknowledgements

We want to thank all the participants for sharing their experiences and the participating primary care centres for support and assistance in conducting the study. We are also grateful to patient research partner Eva Fredholm for constructive discussions throughout the research process.

Authors' contributions

For peer review only - http://bmjopen.bmj.com/site/about/guidelines.xhtml

200522

All authors were involved in the design of the study. LA and SW drafted the manuscript with critical input from EB, AF, HG, EF, KS and IE. IE is the grant holder and project leader. All authors reviewed, edited and approved the final version of the manuscript.

Funding

This work was supported by The Swedish Heart & Lung Foundation (DNr.20180183), the Swedish Research Council (DNr 2017-01230) and the Centre for Person-Centred Care at the University of Gothenburg (GPCC), Sweden. The funders had no role in analysis or preparation of the manuscript.

For peer review only

Competing interests

The authors declare that they have no competing interests.

Patient consent for publication

Because we do not provide personal information in our manuscript, “Consent for publication” is not applicable here.

Data availability statement

The data sets generated and analysed during the current study are not publicly available for ethical reasons but are available from the corresponding author on reasonable request.

Open access

This is an open access article distributed in accordance with the Creative Commons Attribution Non Commercial (CC BY-NC 4.0) license, which permits others to contribute, remix, adapt, build upon this work non-commercially, and license their derivative works on different terms, provided the original work is properly cited, appropriate credit is given, any

For peer review only - http://bmjopen.bmj.com/site/about/guidelines.xhtml

200522

487 changes made indicated and the use is non-commercial (see
488 http://creativecommons.org/licenses/by-nc/4.0/).

489 Word count

490 4766

491

For peer review only

For peer review only - http://bmjopen.bmj.com/site/about/guidelines.xhtml

200522

References

Figure 1 Timeline of the intervention and data collection

1. Rabe KF, Watz H. Chronic obstructive pulmonary disease. *Lancet (London, England)* 2017;389(10082):1931-40. doi: 10.1016/s0140-6736(17)31222-9 [published Online First: 2017/05/18]
2. Ponikowski P, Voors AA, Anker SD, et al. 2016 ESC Guidelines for the Diagnosis and Treatment of Acute and Chronic Heart Failure. *Revista espanola de cardiologia (English ed)* 2016;69(12):1167. doi: 10.1016/j.rec.2016.11.005 [published Online First: 2016/11/30]
3. Theander K, Hasselgren M, Luhr K, et al. Symptoms and impact of symptoms on function and health in patients with chronic obstructive pulmonary disease and chronic heart failure in primary health care. *International journal of chronic obstructive pulmonary disease* 2014;9:785.
4. Holland R, Battersby J, Harvey I, et al. Systematic review of multidisciplinary interventions in heart failure. *Heart* 2005;91(7):899-906.
5. Vogelmeier CF, Criner GJ, Martinez FJ, et al. Global strategy for the diagnosis, management, and prevention of chronic obstructive lung disease 2017 report. GOLD executive summary. *American journal of respiratory and critical care medicine* 2017;195(5):557-82.
6. Wildevuur SE, Simonse LW. Information and communication technology–enabled person-centered care for the “big five” chronic conditions: scoping review. *Journal of medical Internet research* 2015;17(3):e77.
7. Ekman I, Swedberg K, Taft C, et al. Person-centered care—Ready for prime time. *European journal of cardiovascular nursing* 2011;10(4):248-51.
8. Ahmad N, Ellins J, Krelle H, et al. Person-centred care: from ideas to action: Health Foundation 2014.
9. Sen A. Capability and well-being73. *The quality of life* 1993;30
10. Ekman I, Wolf A, Olsson L-E, et al. Effects of person-centred care in patients with chronic heart failure: the PCC-HF study. *European heart journal* 2012;33(9):1112-19.
11. Brännström M, Boman K. Effects of person-centred and integrated chronic heart failure and palliative home care. PREFER: a randomized controlled study. *European journal of heart failure* 2014;16(10):1142-51.
12. Fors A, Ekman I, Taft C, et al. Person-centred care after acute coronary syndrome, from hospital to primary care—a randomised controlled trial. *International journal of cardiology* 2015;187:693-99.
13. Hansson E, Ekman I, Swedberg K, et al. Person-centred care for patients with chronic heart failure - a cost-utility analysis. *European journal of cardiovascular nursing : journal of the Working Group on Cardiovascular Nursing of the European Society of Cardiology* 2016;15(4):276-84. doi: 10.1177/1474515114567035 [published Online First: 2015/01/18]
14. Sahlen KG, Boman K, Brannstrom M. A cost-effectiveness study of person-centered integrated heart failure and palliative home care: Based on a randomized controlled trial. *Palliative medicine* 2016;30(3):296-302. doi: 10.1177/0269216315618544 [published Online First: 2015/11/26]
15. Feldthusen C, Dean E, Forsblad-d'Elia H, et al. Effects of Person-Centered Physical Therapy on Fatigue-Related Variables in Persons With Rheumatoid Arthritis: A Randomized Controlled Trial. *Archives of physical medicine and rehabilitation* 2016;97(1):26-36. doi: 10.1016/j.apmr.2015.09.022 [published Online First: 2015/10/21]

For peer review only

For peer review only - http://bmjopen.bmj.com/site/about/guidelines.xhtml

200522

16. Larsson A, Palstam A, Lofgren M, et al. Resistance exercise improves muscle strength, health status and pain intensity in fibromyalgia--a randomized controlled trial. *Arthritis research & therapy* 2015;17:161. doi: 10.1186/s13075-015-0679-1 [published Online First: 2015/06/19]
17. Fors A, Taft C, Ulin K, et al. Person-centred care improves self-efficacy to control symptoms after acute coronary syndrome: a randomized controlled trial. *European Journal of Cardiovascular Nursing* 2016;15(2):186-94.
18. Wolf A, Vella R, Fors A. The impact of person-centred care on patients' care experiences in relation to educational level after acute coronary syndrome: secondary outcome analysis of a randomised controlled trial. *European journal of cardiovascular nursing : journal of the Working Group on Cardiovascular Nursing of the European Society of Cardiology* 2019;18(4):299-308. doi: 10.1177/1474515118821242 [published Online First: 2019/01/18]
19. Fors A, Gyllensten H, Swedberg K, et al. Effectiveness of person-centred care after acute coronary syndrome in relation to educational level: Subgroup analysis of a two-armed randomised controlled trial. *International journal of cardiology* 2016;221:957-62.
20. Fors A, Swedberg K, Ulin K, et al. Effects of person-centred care after an event of acute coronary syndrome: Two-year follow-up of a randomised controlled trial. *Int J Cardiol* 2017;249:42-47. doi: 10.1016/j.ijcard.2017.08.069 [published Online First: 2017/09/13]
21. Ekman I, Hedman H, Swedberg K, et al. Commentary: Swedish initiative on person centred care. *BMJ (Clinical research ed)* 2015;350:h160. doi: 10.1136/bmj.h160 [published Online First: 2015/02/12]
22. Jansson I, Fors A, Ekman I, et al. Documentation of person-centred health plans for patients with acute coronary syndrome. *European journal of cardiovascular nursing : journal of the Working Group on Cardiovascular Nursing of the European Society of Cardiology* 2018;17(2):114-22. doi: 10.1177/1474515117718415 [published Online First: 2017/06/29]
23. Sanders EB-N, Stappers PJ. Co-creation and the new landscapes of design. *Co-design* 2008;4(1):5-18.
24. Foundation TSI. The swedes and the internet 2019.: Internet Stiftelsen, 2019.
25. Erdelez S, Tanacković SF, Balog KP. Online behavior of the alzheimer's disease patient caregivers on croatian online discussion forum. *Proceedings of the Association for Information Science and Technology* 2019;56(1):78-88.
26. Ali L, Fors A, Ekman I. Need of support in people with chronic obstructive pulmonary disease. *Journal of clinical nursing* 2017
27. Frederix I, Caiani EG, Dendale P, et al. ESC e-Cardiology Working Group Position Paper: Overcoming challenges in digital health implementation in cardiovascular medicine. *European journal of preventive cardiology* 2019;26(11):1166-77.
28. Guo X, Sun Y, Wang N, et al. The dark side of elderly acceptance of preventive mobile health services in China. *Electronic Markets* 2013;23(1):49-61.
29. Hanlon P, Daines L, Campbell C, et al. Telehealth Interventions to Support Self-Management of Long-Term Conditions: A Systematic Metareview of Diabetes, Heart Failure, Asthma, Chronic Obstructive Pulmonary Disease, and Cancer. *J Med Internet Res* 2017;19(5):e172. doi: 10.2196/jmir.6688 [published Online First: 2017/05/21]
30. Fors A, Blanck E, Ali L, et al. Effects of a person-centred telephone-support in patients with chronic obstructive pulmonary disease and/or chronic heart failure–A randomized controlled trial. *PloS one* 2018;13(8):e0203031.
31. Wolf A, Fors A, Ulin K, et al. An eHealth Diary and Symptom-Tracking Tool Combined With Person-Centered Care for Improving Self-Efficacy After a Diagnosis of Acute Coronary Syndrome: A Substudy of a Randomized Controlled Trial. *J Med Internet Res* 2016;18(2):e40. doi: 10.2196/jmir.4890 [published Online First: 2016/02/26]

For peer review only - http://bmjopen.bmj.com/site/about/guidelines.xhtml

32. Chan AW, Tetzlaff JM, Altman DG, et al. SPIRIT 2013 Statement: defining standard protocol items for clinical trials. *Revista panamericana de salud publica = Pan American journal of public health* 2015;38(6):506-14. [published Online First: 2016/07/22]

33. Chan AW, Tetzlaff JM, Gotzsche PC, et al. SPIRIT 2013 explanation and elaboration: guidance for protocols of clinical trials. *BMJ (Clinical research ed)* 2013;346:e7586. doi: 10.1136/bmj.e7586 [published Online First: 2013/01/11]

34. Spinuzzi C. The methodology of participatory design. *Technical Communication* 2005;52(2):163-74.

35. Bandura A. Self-efficacy: toward a unifying theory of behavioral change. *Psychological review* 1977;84(2):191.

36. Lorig KR, Ritter P, Stewart AL, et al. Chronic disease self-management program: 2-year health status and health care utilization outcomes. *Medical care* 2001;39(11):1217-23. doi: 10.1097/00005650-200111000-00008 [published Online First: 2001/10/19]

37. Schwarzer R, Jerusalem M. Generalized self-efficacy scale. *Measures in health psychology: A user's portfolio Causal and control beliefs* 1995;1(1):35-37.

38. Burstrom K, Sun S, Gerdtham UG, et al. Swedish experience-based value sets for EQ-5D health states. *Quality of life research : an international journal of quality of life aspects of treatment, care and rehabilitation* 2014;23(2):431-42. doi: 10.1007/s11136-013-0496-4 [published Online First: 2013/08/27]

39. Zigmond AS, Snaith RP. The hospital anxiety and depression scale. *Acta psychiatrica scandinavica* 1983;67(6):361-70.

40. Ekman I, Granger B, Swedberg K, et al. Measuring shortness of breath in heart failure (SOB-HF): development and validation of a new dyspnoea assessment tool. *European journal of heart failure* 2011;13(8):838-45.

41. Jones P, Brusselle G, Dal Negro R, et al. Properties of the COPD assessment test in a cross-sectional European study. *European Respiratory Journal* 2011;38(1):29-35.

42. Stenton C. The MRC breathlessness scale. *Occupational Medicine* 2008;58(3):226-27.

43. Dolan P, Gudex C, Kind P, et al. A social tariff for EuroQol: results from a UK general population survey, 1995.

44. Segel JE. Cost-of-illness studies—a primer. *RTI-UNC Center of Excellence in Health Promotion Economics* 2006:1-39.

45. Drummond MF, Drummond MF, McGuire A. Economic evaluation in health care: merging theory with practice: OUP Oxford 2001.

46. Craig P, Dieppe P, Macintyre S, et al. Developing and evaluating complex interventions: the new Medical Research Council guidance. *BMJ (Clinical research ed)* 2008;337:a1655. doi: 10.1136/bmj.a1655 [published Online First: 2008/10/01]

47. Moore GF, Audrey S, Barker M, et al. Process evaluation of complex interventions: Medical Research Council guidance. *BMJ (Clinical research ed)* 2015;350:h1258.

48. Charmaz K. Constructing grounded theory: sage 2014.

49. Fetters MD, Curry LA, Creswell JW. Achieving integration in mixed methods designs—principles and practices. *Health services research* 2013;48(6pt2):2134-56.

50. Krueger RA. Focus groups: A practical guide for applied research: Sage publications 2014.

51. Graneheim UH, Lundman B. Qualitative content analysis in nursing research: concepts, procedures and measures to achieve trustworthiness. *Nurse education today* 2004;24(2):105-12.

52. World Medical Association Declaration of Helsinki: ethical principles for medical research involving human subjects. *Jama* 2013;310(20):2191-4. doi: 10.1001/jama.2013.281053 [published Online First: 2013/10/22]

53. Campbell NC, Murray E, Darbyshire J, et al. Designing and evaluating complex interventions to improve health care. *BMJ (Clinical research ed)* 2007;334(7591):455-59.

For peer review only

For peer review only - http://bmjopen.bmj.com/site/about/guidelines.xhtml

200522

54. Inglis SC, Clark RA, Dierckx R, et al. Structured telephone support or non-invasive telemonitoring for patients with heart failure. *Cochrane Database of Systematic Reviews* 2015(10)
55. Alfredsson J, Alexander KP. Multiple chronic conditions in older adults with acute coronary syndromes. *Clinics in geriatric medicine* 2016;32(2):291-303.
56. Dharmarajan K, Strait KM, Tinetti ME, et al. Treatment for multiple acute cardiopulmonary conditions in older adults hospitalized with pneumonia, chronic obstructive pulmonary disease, or heart failure. *Journal of the American Geriatrics Society* 2016;64(8):1574-82.
57. Hawkins NM, Petrie MC, Jhund PS, et al. Heart failure and chronic obstructive pulmonary disease: diagnostic pitfalls and epidemiology. *Eur J Heart Fail* 2009;11(2):130-9. doi: 10.1093/eurjhf/hfn013 [published Online First: 2009/01/27]

For peer review only

For peer review only - http://bmjopen.bmj.com/site/about/guidelines.xhtml

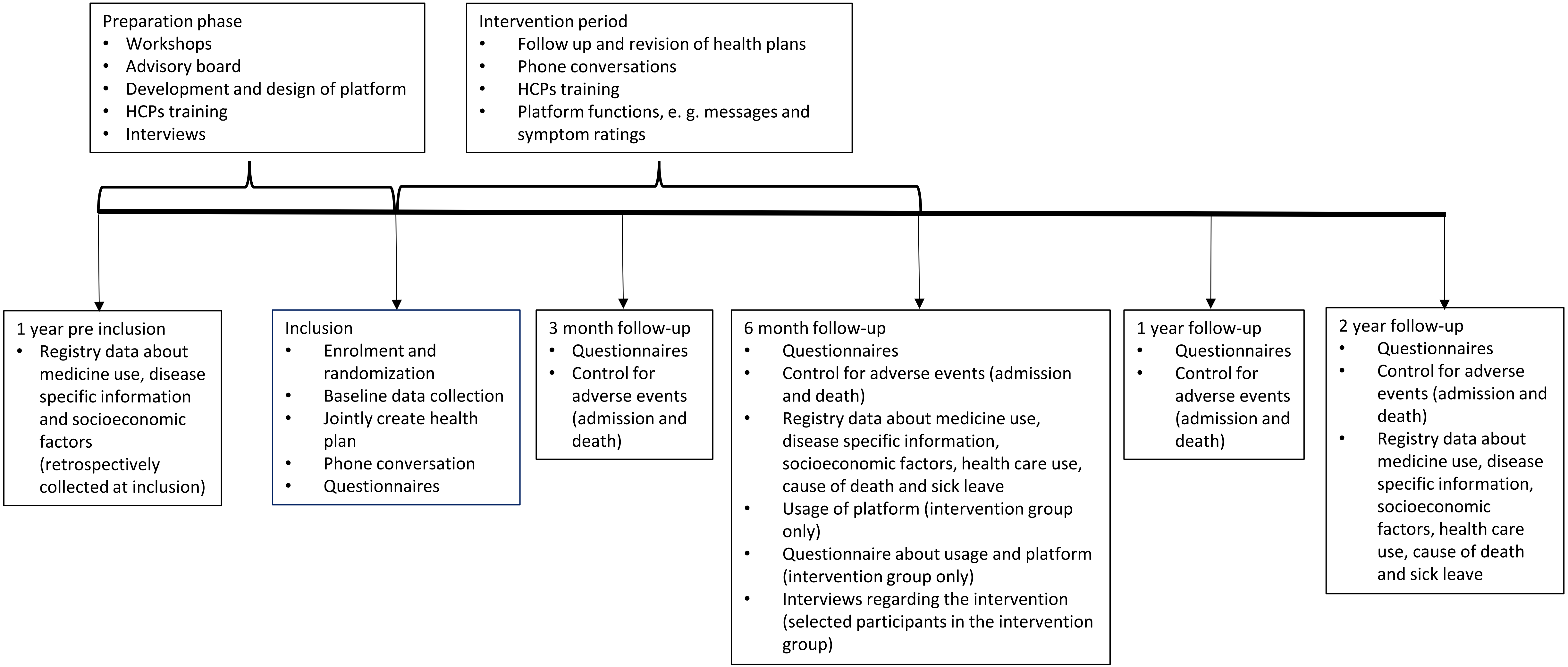
Preparation phase
• Workshops
• Advisory board
• Development and design of platform
• HCPs training
• Interviews
Intervention period
• Follow up and revision of health plans
• Phone conversations
• HCPs training
• Platform functions, e. g. messages and symptom ratings
1 year pre inclusion
• Registry data about medicine use, disease specific information and socioeconomic factors (retrospectively collected at inclusion)
Inclusion
• Enrolment and randomization
• Baseline data collection
• Jointly create health plan
• Phone conversation
• Questionnaires
3 month follow-up
• Questionnaires
• Control for adverse events (admission and death)
6 month follow-up
• Questionnaires
• Control for adverse events (admission and death)
• Registry data about medicine use, disease specific information, socioeconomic factors, health care use, cause of death and sick leave
• Usage of platform (intervention group only)
• Questionnaire about usage and platform (intervention group only)
• Interviews regarding the intervention (selected participants in the intervention group)
1 year follow-up
• Questionnaires
• Control for adverse events (admission and death)
2 year follow-up
• Questionnaires
• Control for adverse events (admission and death)
• Registry data about medicine use, disease specific information, socioeconomic factors, health care use, cause of death and sick leave

Reporting checklist for protocol of a clinical trial.

Based on the SPIRIT guidelines.

Instructions to authors

Complete this checklist by entering the page numbers from your manuscript where readers will find each of the items listed below.

Your article may not currently address all the items on the checklist. Please modify your text to include the missing information. If you are certain that an item does not apply, please write "n/a" and provide a short explanation.

Upload your completed checklist as an extra file when you submit to a journal.

In your methods section, say that you used the SPIRITreporting guidelines, and cite them as:

Chan A-W, Tetzlaff JM, Altman DG, Laupacis A, Gøtzsche PC, Krleža-Jerić K, Hróbjartsson A, Mann H, Dickersin K, Berlin J, Doré C, Parulekar W, Summerskill W, Groves T, Schulz K, Sox H, Rockhold FW, Rennie D, Moher D. SPIRIT 2013 Statement: Defining standard protocol items for clinical trials. Ann Intern Med. 2013;158(3):200-207

		Reporting Item	Page Number
**Administrative information**			
Title	#1	Descriptive title identifying the study design, population, interventions, and, if applicable, trial acronym	1
Trial registration	#2a	Trial identifier and registry name. If not yet registered, name of intended registry	2
Trial registration: data set	#2b	All items from the World Health Organization Trial Registration Data Set	2
Protocol version	#3	Date and version identifier	Top of page
Funding	#4	Sources and types of financial, material, and other support	23
Roles and	#5a	Names, affiliations, and roles of protocol contributors	1,23

For peer review only

responsibilities: contributorship			
Roles and responsibilities: sponsor contact information	#5b	Name and contact information for the trial sponsor	23
Roles and responsibilities: sponsor and funder	#5c	Role of study sponsor and funders, if any, in study design; collection, management, analysis, and interpretation of data; writing of the report; and the decision to submit the report for publication, including whether they will have ultimate authority over any of these activities	23
Roles and responsibilities: committees	#5d	Composition, roles, and responsibilities of the coordinating centre, steering committee, endpoint adjudication committee, data management team, and other individuals or groups overseeing the trial, if applicable (see Item 21a for data monitoring committee)	N/A
**Introduction**			
Background and rationale	#6a	Description of research question and justification for undertaking the trial, including summary of relevant studies (published and unpublished) examining benefits and harms for each intervention	4-7
Background and rationale: choice of comparators	#6b	Explanation for choice of comparators	4-7
Objectives	#7	Specific objectives or hypotheses	7
Trial design	#8	Description of trial design including type of trial (eg, parallel group, crossover, factorial, single group), allocation ratio, and framework (eg, superiority, equivalence, non-inferiority, exploratory)	8
**Methods: Participants, interventions, and outcomes**			

For peer review only

Study setting	#9	Description of study settings (eg, community clinic, academic hospital) and list of countries where data will be collected. Reference to where list of study sites can be obtained	8-9
Eligibility criteria	#10	Inclusion and exclusion criteria for participants. If applicable, eligibility criteria for study centres and individuals who will perform the interventions (eg, surgeons, psychotherapists)	9
Interventions: description	#11a	Interventions for each group with sufficient detail to allow replication, including how and when they will be administered	10-13
Interventions: modifications	#11b	Criteria for discontinuing or modifying allocated interventions for a given trial participant (eg, drug dose change in response to harms, participant request, or improving / worsening disease)	10-13
Interventions: adherance	#11c	Strategies to improve adherence to intervention protocols, and any procedures for monitoring adherence (eg, drug tablet return; laboratory tests)	N/A
Interventions: concomitant care	#11d	Relevant concomitant care and interventions that are permitted or prohibited during the trial	N/A
Outcomes	#12	Primary, secondary, and other outcomes, including the specific measurement variable (eg, systolic blood pressure), analysis metric (eg, change from baseline, final value, time to event), method of aggregation (eg, median, proportion), and time point for each outcome. Explanation of the clinical relevance of chosen efficacy and harm outcomes is strongly recommended	13-14
Participant timeline	#13	Time schedule of enrolment, interventions (including any run-ins and washouts), assessments, and visits for participants. A schematic diagram is highly recommended (see Figure)	15 + figure
Sample size	#14	Estimated number of participants needed to achieve study objectives and how it was determined, including clinical and statistical assumptions supporting any sample size calculations	15

For peer review only

Recruitment	#15	Strategies for achieving adequate participant enrolment to reach target sample size	9-10
**Methods: Assignment of interventions (for controlled trials)**			
Allocation: sequence generation	#16a	Method of generating the allocation sequence (eg, computer-generated random numbers), and list of any factors for stratification. To reduce predictability of a random sequence, details of any planned restriction (eg, blocking) should be provided in a separate document that is unavailable to those who enrol participants or assign interventions	9-10
Allocation concealment mechanism	#16b	Mechanism of implementing the allocation sequence (eg, central telephone; sequentially numbered, opaque, sealed envelopes), describing any steps to conceal the sequence until interventions are assigned	9-10
Allocation: implementation	#16c	Who will generate the allocation sequence, who will enrol participants, and who will assign participants to interventions	9-10
Blinding (masking)	#17a	Who will be blinded after assignment to interventions (eg, trial participants, care providers, outcome assessors, data analysts), and how	9-10
Blinding (masking): emergency unblinding	#17b	If blinded, circumstances under which unblinding is permissible, and procedure for revealing a participant's allocated intervention during the trial	N/A
**Methods: Data collection, management, and analysis**			
Data collection plan	#18a	Plans for assessment and collection of outcome, baseline, and other trial data, including any related processes to promote data quality (eg, duplicate measurements, training of assessors) and a description of study instruments (eg, questionnaires, laboratory tests)	13-14

For peer review only

		along with their reliability and validity, if known. Reference to where data collection forms can be found, if not in the protocol	
Data collection plan: retention	#18b	Plans to promote participant retention and complete follow-up, including list of any outcome data to be collected for participants who discontinue or deviate from intervention protocols	13-14
Data management	#19	Plans for data entry, coding, security, and storage, including any related processes to promote data quality (eg, double data entry; range checks for data values). Reference to where details of data management procedures can be found, if not in the protocol	19-20
Statistics: outcomes	#20a	Statistical methods for analysing primary and secondary outcomes. Reference to where other details of the statistical analysis plan can be found, if not in the protocol	13-19
Statistics: additional analyses	#20b	Methods for any additional analyses (eg, subgroup and adjusted analyses)	13-19
Statistics: analysis population and missing data	#20c	Definition of analysis population relating to protocol non-adherence (eg, as randomised analysis), and any statistical methods to handle missing data (eg, multiple imputation)	13-19
**Methods: Monitoring**			
Data monitoring: formal committee	#21a	Composition of data monitoring committee (DMC); summary of its role and reporting structure; statement of whether it is independent from the sponsor and competing interests; and reference to where further details about its charter can be found, if not in the protocol. Alternatively, an explanation of why a DMC is not needed	N/A
Data monitoring: interim analysis	#21b	Description of any interim analyses and stopping guidelines, including who will have access to these interim results and make the final decision to terminate the trial	N/A
Harms	#22	Plans for collecting, assessing, reporting, and managing solicited and spontaneously reported adverse events and	13-19 + Figure

For peer review only

		other unintended effects of trial interventions or trial conduct	
Auditing	#23	Frequency and procedures for auditing trial conduct, if any, and whether the process will be independent from investigators and the sponsor	N/A
**Ethics and dissemination**			
Research ethics approval	#24	Plans for seeking research ethics committee / institutional review board (REC / IRB) approval	19-20
Protocol amendments	#25	Plans for communicating important protocol modifications (eg, changes to eligibility criteria, outcomes, analyses) to relevant parties (eg, investigators, REC / IRBs, trial participants, trial registries, journals, regulators)	N/A
Consent or assent	#26a	Who will obtain informed consent or assent from potential trial participants or authorised surrogates, and how (see Item 32)	N/A
Consent or assent: ancillary studies	#26b	Additional consent provisions for collection and use of participant data and biological specimens in ancillary studies, if applicable	N/A
Confidentiality	#27	How personal information about potential and enrolled participants will be collected, shared, and maintained in order to protect confidentiality before, during, and after the trial	19-20
Declaration of interests	#28	Financial and other competing interests for principal investigators for the overall trial and each study site	23
Data access	#29	Statement of who will have access to the final trial dataset, and disclosure of contractual agreements that limit such access for investigators	23
Ancillary and post trial care	#30	Provisions, if any, for ancillary and post-trial care, and for compensation to those who suffer harm from trial participation	N/A
Dissemination policy: trial results	#31a	Plans for investigators and sponsor to communicate trial results to participants, healthcare professionals, the public, and other relevant groups (eg, via publication,	19-20

For peer review only

		reporting in results databases, or other data sharing arrangements), including any publication restrictions	
Dissemination policy: authorship	#31b	Authorship eligibility guidelines and any intended use of professional writers	N/A
Dissemination policy: reproducible research	#31c	Plans, if any, for granting public access to the full protocol, participant-level dataset, and statistical code	23

Appendices

Informed consent materials	#32	Model consent form and other related documentation given to participants and authorised surrogates	Appendix
Biological specimens	#33	Plans for collection, laboratory evaluation, and storage of biological specimens for genetic or molecular analysis in the current trial and for future use in ancillary studies, if applicable	N/A

None The SPIRIT checklist is distributed under the terms of the Creative Commons Attribution License CC-BY-ND 3.0. This checklist can be completed online using https://www.goodreports.org/, a tool made by the EQUATOR Network in collaboration with Penelope.ai

For peer review only
